# Mechanically Adaptive Polyimide Interfaces for Stable High‐Voltage NCM–Sulfide All‐Solid‐State Batteries

**DOI:** 10.1002/advs.75595

**Published:** 2026-05-08

**Authors:** Jiatao Wu, Wenjin Li, Rui Wang, Peng Wang, Kaiyuan Deng, Chengshuai Chang, Chuan Xie, Lei Yao, Guangliang Gary Liu

**Affiliations:** ^1^ Guangdong Provincial Key Laboratory of New Energy Materials Service Safety College of Materials Science and Engineering Shenzhen University Shenzhen China; ^2^ Faculty of Civil Aviation and Aeronautics Kunming University of Science and Technology Kunming China

**Keywords:** all‐solid‐state battery, interfacial engineering, nickel‐rich cathode, polyimide coating

## Abstract

The practical implementation of Ni‐rich layered oxide–sulfide all‐solid‐state batteries (ASSBs) is limited by rapid capacity fading from interfacial contact loss and parasitic reactions. Conventional inorganic coatings provide limited mechanical compliance, failing to address these intertwined electrochemical–mechanical degradations. Here, we report a smart responsive cathode–electrolyte interface realized through the stepwise construction of a conformal polyimide (PI) coating on single‐crystal LiNi_0.8_Co_0.1_Mn_0.1_O_2_ (sNCM). The grafted PI layer establishes robust carboxylate–transition metal coordination bonds, effectively eliminating surface lithium residues and suppressing oxygen release. Owing to its viscoelastic nature, the PI coating reduces the surface modulus of sNCM by 26.6%, accommodating anisotropic volume changes and mitigating intragranular microcrack propagation. Most significantly, the PI interface functions as an electrochemically driven self‐optimizing system during cycling, progressively decreasing total interfacial impedance by 38%. Consequently, the sNCM@PI0.05 cathode delivers 83.6% capacity retention after 400 cycles at 1C under a 4.3 V cutoff voltage, significantly outperforming unmodified sNCM (33.2% retention). It also maintains 90.2% of its initial capacity after 200 cycles at 4.5 V. This reactive polymer interphase design harmonizes chemical passivation and mechanical adaptability, providing a transformative strategy for durable, high‐energy ASSBs.

## Graphical abstract

A mechanically adaptive polyimide interface dynamically stabilizes single‐crystal cathodes in sulfide all‐solid‐state batteries. By integrating robust chemical coordination with viscoelastic compliance, this smart coating suppresses parasitic reactions and accommodates cyclic volume expansion. The resulting self‐optimizing interface mitigates chemo‐mechanical degradation, unlocking exceptional high‐voltage cycling stability for solid‐state storage.

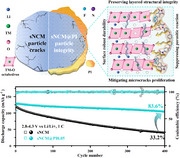

## 1 Introduction

All‐solid‐state lithium batteries (ASSBs) represent a transformative energy storage paradigm for electric vehicles and grid‐scale applications, offering intrinsic safety through elimination of flammable liquid electrolytes while enabling unprecedented energy densities [1, 2]. Among various ASSB configurations, the integration of high‐nickel layered oxides LiNi_x_Co_y_Mn_1‐x‐y_O_2_ (NCM) with sulfide‐based solid electrolytes, such as Li_6_PS_5_Cl (LPSC), has emerged as a promising archetype. Specifically, single‐crystal NCM811 (sNCM) delivers exceptional capacity (>200 mAh g^−1^) through reversible Li^+^ (de)intercalation, while LPSC provides ultrahigh ionic conductivity (>10^−3^ S cm^−1^) and mechanical compliance for intimate interfacial contact [3, 4, 5, 6]. Despite this synergistic pairing, practical implementation remains critically constrained by rapid capacity decay during extended cycling, especially under high‐voltage operation.

The issue of rapid capacity fading primarily arises from two interrelated challenges. During deep cycling, sNCM undergoes anisotropic lattice contraction/expansion during H2→H3 phase transitions, generating substantial internal stresses that propagate intragranular microcracks even within single‐crystal architectures [7, 8]. Unlike liquid electrolytes that can penetrate such defects, rigid thiophosphate solid‐state electrolytes (SSE) cannot refill these emerging voids, leading to progressive contact loss and active material isolation [9]. On the other hand, the formation of undesired interfacial by‐products further accelerates performance degradation. Upon delithiation, strong Ni 3d‐O 2p hybridization weakens transition metal‐oxygen bonds, promoting the release of reactive oxygen species [10, 11]. This instability promotes the release of reactive oxygen species (e.g., O_n‐_, O_2_), attacking LPSC via O‐S exchange reactions to generate insulating interfacial by‐products [12, 13]. These two degradation pathways are reciprocally coupled through a self‐amplifying feedback loop: micro‐crack propagation continually unveils pristine cathode surfaces to electrolyte assault, while the resultant interfacial by‐products conversely elevate local mechanical stress intensity, thereby accelerating crack tip driving forces [1, 4, 14].

Conventional modification strategies employing inorganic coatings (e.g., LiNbO_3_, LiTaO_3_, LiZr_2_(PO_4_)_3_) exhibit intrinsic limitations in addressing this dual challenge [15, 16, 17]. The high Young's modulus (e.g., LiNbO_3_ ∼195 GPa) renders them brittle under cyclic mechanical stress, leading to coating fracture and delamination. Moreover, their chemical inertness provides incomplete suppression of O‐S exchange reactions due to poor accommodation of dynamic interfacial reconstruction during (de)lithiation. While organic polymer coatings generally exhibit improved mechanical compliance compared to their inorganic counterparts, they still suffer from critical deficiencies when addressing the complex electro‐chemo‐mechanical degradation [18, 19, 20, 21, 22, 23]. For instance, conventional polyvinylidene fluoride relies primarily on weak van der Waals interactions, lacking the robust chemical anchoring necessary to maintain interfacial integrity during cyclic lattice expansion [24, 25]. Similarly, although polyacrylic acid offers strong interfacial bonding via abundant carboxyl groups [26, 27], its dense hydrogen‐bonding network renders it mechanically rigid, thus lacking the viscoelasticity required to buffer dynamic volume changes. Furthermore, conjugated conductive polymers like PEDOT:PSS provide efficient charge percolation networks [28, 29], but lack specific chemical scavenging capabilities and dynamic structural adaptability, limiting their ability to undergo favorable interfacial activation during cycling. Consequently, these materials often address only isolated aspects of the complex interfacial challenges, failing to simultaneously mitigate mechanical degradation and chemical side reactions under stringent operating conditions.

**SCHEME 1:**
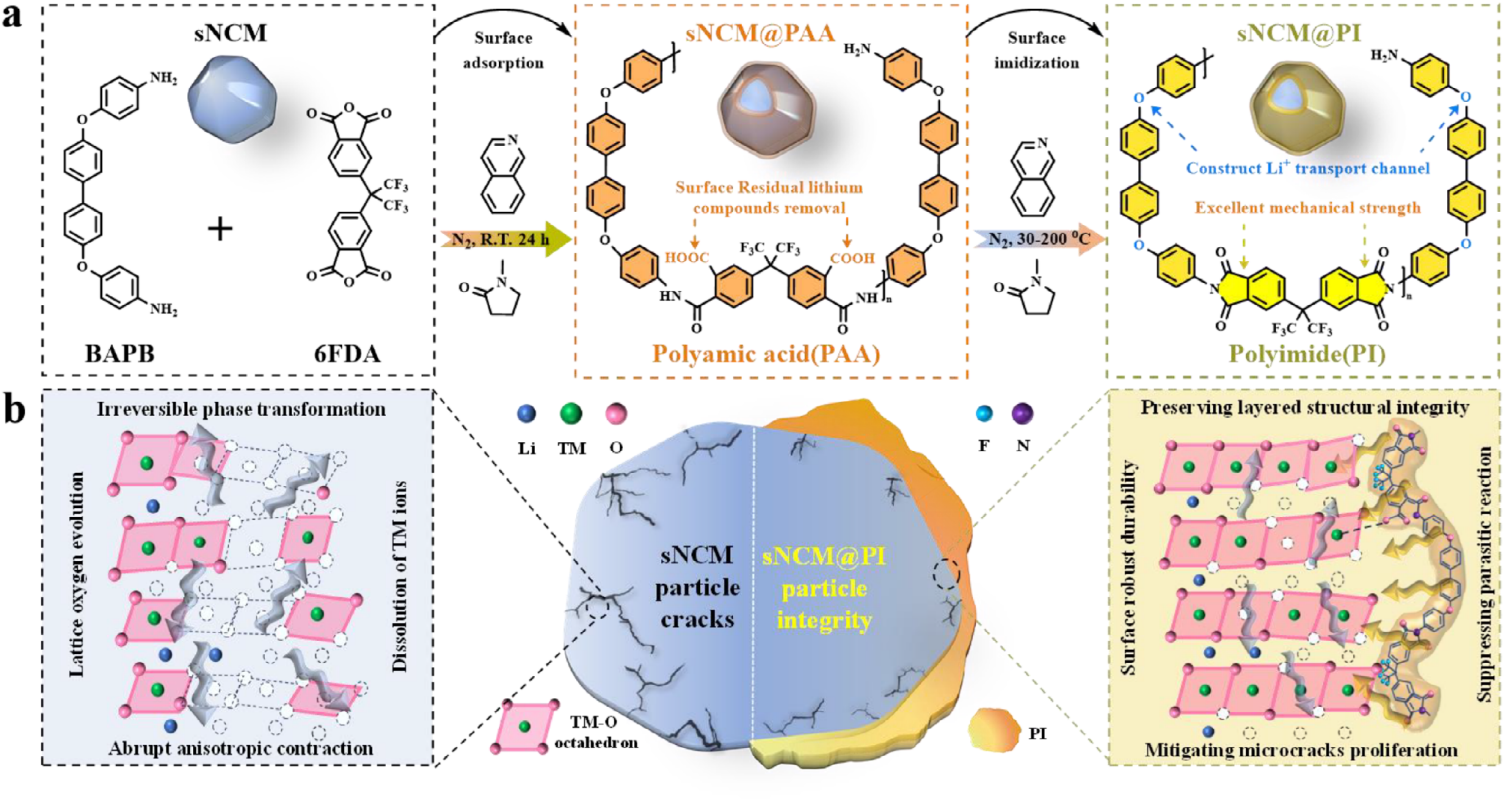
(a) Schematic illustration of the fabrication process for PI‐coated sNCM (sNCM@PI). (b) The schematic illustration of deterioration for sNCM and sNCM@PI cathodes during cycling.

To overcome these isolated limitations, it is highly desirable to develop a protective layer that synergistically integrates a triad of functionalities: chemical anchoring, viscoelasticity, and electrochemical activation. Herein, we put forward a rational interfacial architecture to achieve a smart responsive interface through nano‐conformal polyimide (PI) coating grafted onto sNCM (Scheme 1). Through precise polycondensation of 4,4'‐(hexafluoroisopropylidene)diphthalic anhydride (6FDA) and 1,3‐bis(3‐aminophenoxy)benzene (BAPB), the PI coating forms robust carboxylate‐transition metal coordination bonds with the cathode surface. This chemical bonding mechanism effectively eliminates residual lithium species (Li_2_CO_3_/LiOH), therefore creating a chemically passivated interface that suppresses oxygen release and subsequent electrolyte decomposition. Furthermore, the viscoelastic nature of PI (surface modulus reduced by 26.6% from 9.76 to 7.16 GPa) enables dynamic accommodation of anisotropic volume changes during cycling, preventing microcrack initiation and maintaining continuous cathode‐electrolyte contact. Notably, unlike conventional static protective layers [9, 18, 27, 30, 31, 32, 33, 34, 35, 36, 37, 38, 39], the PI interface may undergo a unique electrochemically activated dynamic self‐optimization during cycling. This smart responsive behavior is manifested as a remarkable 38% reduction in total interfacial impedance, offering an alternative to traditional coatings which often experience degradation during extended cycling. The optimized sNCM@PI0.05 cathode delivers exceptional cycling stability, retaining 83.6% of its initial capacity after 400 cycles at 1C, significantly outperforming the unmodified sNCM (33.2%). Even at a high cut‐off voltage of 4.5 V, the modified cathode maintains its robust electrochemical stability, delivering a capacity retention of 90.2% after 200 cycles. Subsequent characterization reveals that this performance stems from the triple functionality of the PI coating, which simultaneously suppresses interfacial side reactions, mitigates microcrack proliferation, and autonomously optimizes interfacial ion transport through electrochemical activation. This work establishes PI as a dynamic interfacial mediator that harmonizes chemical passivation with mechanical adaptability while evolving toward optimal performance. This approach pioneers a new design philosophy for next‐generation ASSBs where interfaces are not merely protective barriers but intelligent, adaptive systems that grow stronger with use.

## 2 Results and Discussion

### 2.1 Materials Characterizations

**FIGURE 1:**
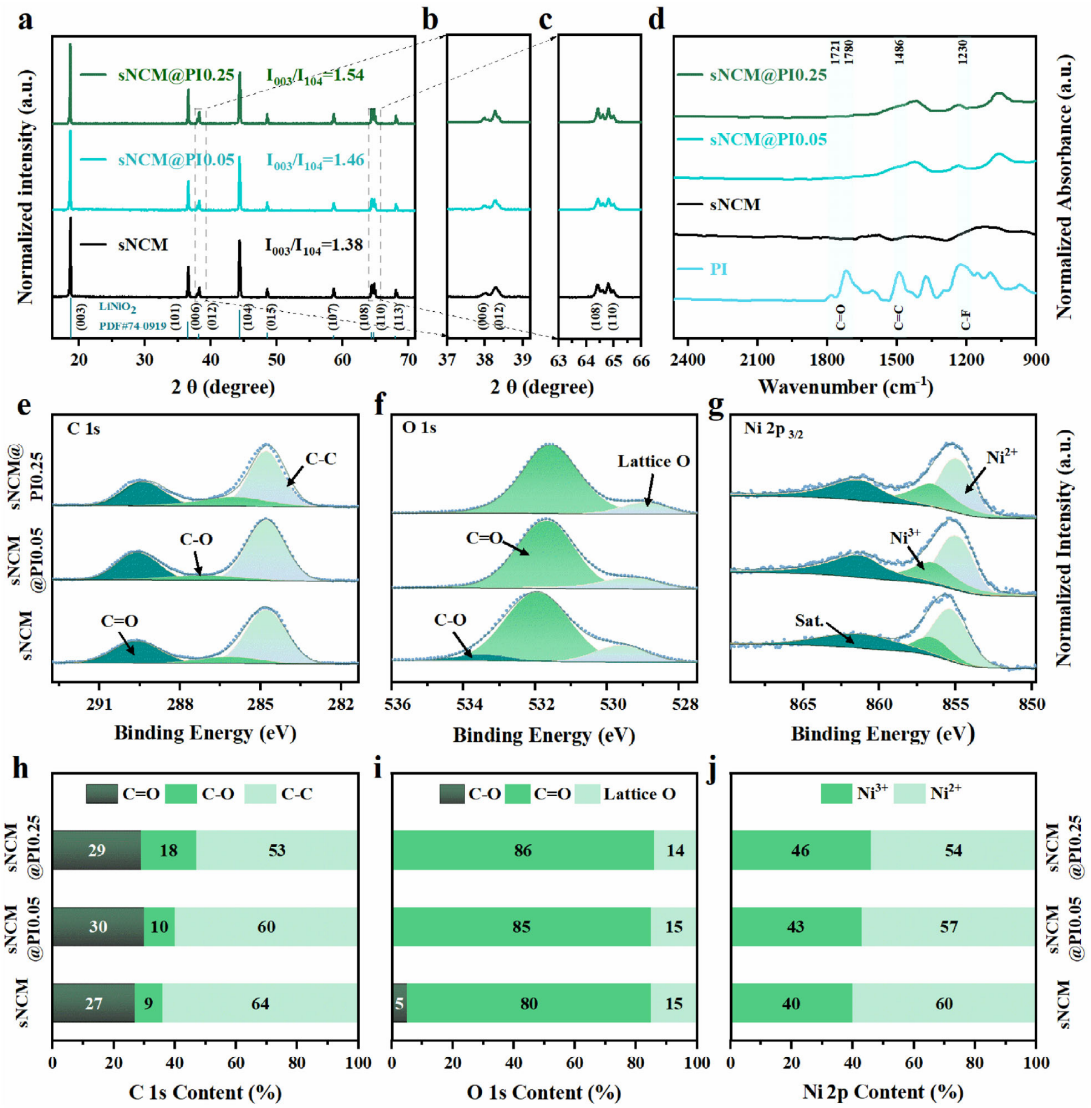
(a) XRD patterns of sNCM, sNCM@PI0.05, and sNCM@PI0.25. (b,c) Partially magnified regions of (a). (d) FT‐IR spectra of pristine PI, sNCM, sNCM@PI0.05, and sNCM@PI0.25. (e–g) XPS spectra of C 1s (e), O 1s (f), and Ni 2p (g) for sNCM, sNCM@PI0.05, and sNCM@PI0.25, respectively, together with their corresponding deconvoluted component peaks shown in (h–j).

The sNCM@PI composite cathode was fabricated through a three‐step procedure involving the synthesis of poly(amic acid) (PAA), adsorption of PAA onto the sNCM surface, and subsequent stepwise thermal imidization to cyclize the adsorbed PAA into a conformal 6FDA–BAPB PI coating (Figure S1). The synthesis of the 6FDA–BAPB PI was confirmed by gel permeation chromatography (GPC) and Fourier‐transform infrared (FT‐IR) spectroscopy. GPC analysis (Figure S2) indicated a number‐average molecular weight of 51 kDa and a polydispersity index of 1.56, consistent with a well‐defined step‐growth polymerization mechanism [2, 3, 4, 5]. FT‐IR spectroscopy (Figure 1d) further corroborated the formation of the imide structure, as evidenced by characteristic vibrational bands at 1780 and 1721 cm^−1^ (asymmetric and symmetric C = O stretches), 1486 cm^−1^ (aromatic C = C), and 1230 cm^−1^ (C–F deformation) [6, 24, 40, 41, 42]. X‐ray diffraction (XRD) patterns (Figure 1a–c) confirmed that all samples maintained a well‐ordered α‐NaFeO_2_‐type layered structure (space group *R^−^3m*) [12]. The pristine sNCM exhibits an I_(003)_/I_(104)_ intensity ratio of 1.38, indicative of moderate Li^+^/Ni^2+^ cation mixing. After PI modification, the I_(003)_/I_(104)_ ratios increased systematically to 1.46 and 1.54 for sNCM@PI0.05 and sNCM@PI0.25, respectively, reflecting an improvement in cationic ordering [13, 43]. The distinct splitting of the (006)/(012) and (108)/(110) peak pairs confirmed that the coating process preserves the host crystalline structure without inducing significant lattice strain [36, 44]. The presence of a conformal PI was further supported by FT‐IR, which showed the same characteristic imide peaks on coated samples that were absent in pristine sNCM (Figure 1d). Thermogravimetric analysis (TGA, Figure S3) revealed a progressive increase in mass loss with PI content, corroborating controlled loading.

X‐ray photoelectron spectroscopy (XPS) reveals critical interfacial interactions at the PI‐sNCM junction, demonstrating that the PI overlayer functions not only as a physical barrier but also as a chemical modifier of the cathode surface. In the C 1s region (Figure 1h), the C = O component intensifies from 27% in pristine sNCM to 30% and 28% for sNCM@PI0.05 and sNCM@PI0.25, respectively, while the C‐C peak correspondingly declines from 64% to 60% and 53%, respectively. This systematic transformation, quantified by a rise in the C = O/C‐C ratio from 0.42 to approximately 0.54, supports the establishment of a PI‐dominated surface chemistry [24, 45, 46]. Notably, the PI coating induces a restructuring of the interfacial oxygen environment as demonstrated in Figure 1f. For the pristine sNCM, the O 1s spectrum can be deconvoluted into lattice oxygen (lattice O, 529.53 eV), surface carbonates (C = O, 531.98 eV), and adventitious C‐O/LiOH residues (533.50 eV) [21, 47, 48, 49, 50]. Upon PI coating, the parasitic C‐O peak diminishes to negligible levels. Meanwhile, the C = O component increases to 85% due to the abundant carbonyl groups inherent to PI, accompanied by a shift to lower binding energies (from 531.98 to 531.55 eV). This concurrent elimination of residue signals and systematic binding energy shift supports the effective clearance of parasitic species and surface passivation [21, 51, 52]. Intriguingly, the C 1s signal of carboxyl also shifts to a higher BE (from 288.55 eV to 289.36 eV) after PI coating (Table S1). These shifts aligned with the formation of carboxylate‐transition metal bonds, which likely arise from reactions between surface Ni/Li species and PAA carboxylic groups during imidization (proposed mechanism in Figure S4) [21, 24, 45, 49, 53, 54]. Following PI coating, the sNCM cathode is encapsulated by the coating layer. Due to the surface‐sensitive nature of XPS, the detected proportion of lattice oxygen decreases marginally from 15% to 14% [35, 49]. In addition, the Ni 2p spectra show a systematic increase in the Ni^3+^/Ni^2+^ ratio from 0.67 (sNCM) to 0.85 (sNCM@PI0.25). This increase indicates the suppression of Ni^3+^ reduction and cation disordering, which positively contributes to the observed enhancements in electrochemical performance. Together, these results establish that the PI overlayer acts synergistically as a physical barrier and a chemical stabilizer, propagating interfacial benefits into the bulk lattice to enhance structural order.

**FIGURE 2:**
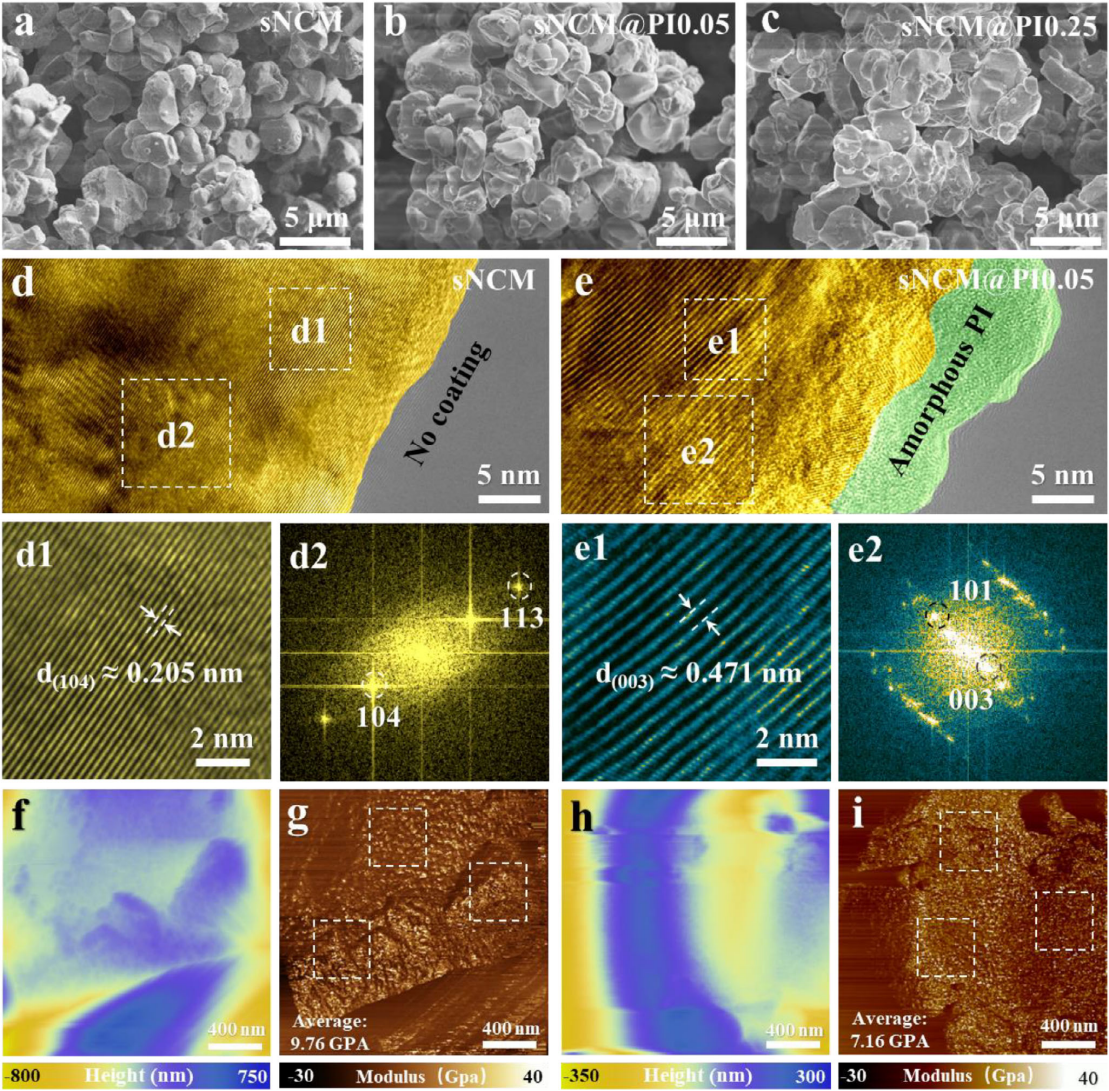
SEM images of (a) sNCM, (b) sNCM@PI0.05, and (c) sNCM@PI0.25 cathodes. HR‐TEM images of (d) sNCM (images d1 and d2 corresponding to position d1 and d2 in d) and (e) sNCM@PI0.05 (images e1 and e2 corresponding to position e1 and e2 in e). AFM images for the surface morphology of (f) sNCM and (h) sNCM@PI0.05 and the corresponding Young's modulus of (g) sNCM and (i) sNCM@PI0.05.

Building on XPS‐confirmed suppression of residual lithium species, morphological characterization further elucidates the structure‐property relationships in PI‐coated sNCM. Scanning electron microscopy (SEM) revealed no discernible alteration in particle morphology after PI coating (Figure 2a–c; Figure S5). However, excessive loading (sNCM@PI0.25) induced surface roughness which likely elevated interfacial impedance, a critical factor for governing Li^+^ transport in solid‐state systems (Figure S5) [55, 56]. Therefore, sNCM@PI0.05 was prioritized for its optimized interfacial architecture. High‐resolution transmission electron microscopy (HR‐TEM) demonstrated that pristine sNCM exhibited smooth surfaces with (104) lattice fringes (0.205 nm; Figure 2d), while sNCM@PI0.05 was encapsulated by a uniform amorphous PI layer (∼4.0 nm thick) that preserved the underlying (003) lattice spacing (0.471 nm; Figure 2e) [39, 57]. Energy‐dispersive spectroscopy (EDS) mappings (Figures S6–S8) confirmed homogeneous coating coverage, as demonstrated by the spatial overlap of C, F, and N signals with those of the transition metals (Ni, Co, Mn). Critically, atomic force microscopy (AFM) demonstrated a 26.6% reduction in surface modulus (from 9.76 to 7.16 GPa; Figure 2i), suggesting improved strain accommodation during cycling. To further assess the bulk mechanical properties, nanoindentation was employed on pressed composite cathode pellets of sNCM and sNCM@PI0.05 [58, 59]. As depicted in Figure S9, the PI coating induced a substantial mechanical modulation, manifested by a marked reduction in the bulk Young's modulus from 8.51 ± 0.8 GPa for sNCM to 1.34 ± 0.5 GPa for sNCM@PI0.05. Although absolute values diverge from AFM measurements owing to differences in probing depth and contact mechanics [60], both techniques consistently corroborate the enhanced mechanical compliance of the coated cathode. This synergistic viscoelastic buffering, operating across both nanoscale and mesoscale regimes, is pivotal for preserving structural integrity under dynamic electro‐chemo‐mechanical stresses [61, 62, 63, 64]. Consequently, the PI layer can effectively suppress residual lithium compounds and stabilize the cathode‐electrolyte interface. The dual functionality positions the sNCM@PI composite as a promising candidate for enhanced solid‐state battery performance, as evaluated in the subsequent section [55, 65].

### 2.2 Electrochemical Performance of sNCM@PI‐Based ASSBs

**FIGURE 3:**
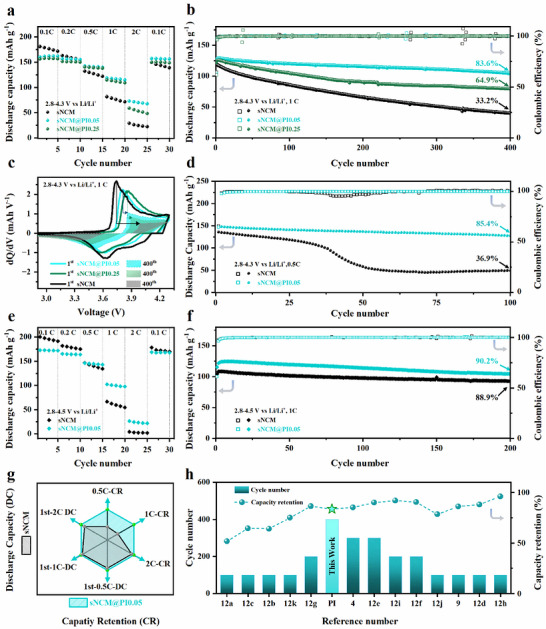
Electrochemical performance of pristine sNCM, sNCM@PI0.05, and sNCM@PI0.25. (a) Rate performance, (b) long‐term cycling at 1C, and (c) differential capacity (*dQ/dV*) profiles at 1C of sNCM, sNCM@PI0.05, and sNCM@PI0.25 in the voltage range of 2.8‐4.3 V vs Li/Li^+^ at 25°C. (d) Long‐term cycling at 0.5C of sNCM and sNCM@PI0.05 in the voltage range of 2.8–4.3 V vs Li/Li^+^ at 25°C. (e) Rate performance and (f) long‐term cycling at 1C of sNCM and sNCM@PI0.05 within the voltage range of 2.8–4.5 V vs Li/Li^+^ at 25°C. (g) Radar chart comparing key electrochemical metrics of sNCM and sNCM@PI0.05. (h) Long‐term cycling performance of sNCM@PI0.05 versus state‐of‐the‐art modified NCM cathodes reported in literature [9, 18, 27, 30, 31, 32, 33, 34, 35, 36, 37, 38, 39].

To evaluate the effectiveness of PI surface coating on the electrochemical performance of sNCM, ASSBs were assembled using pristine sNCM and PI‐coated sNCM cathodes, a commercial sulfide electrolyte (LPSC), and a Li‐In alloy anode. The Li‐In alloy was employed as the anode to mitigate the thermodynamic instability, kinetic limitations, and mechanical contact loss typically associated with pure Li metal, ensuring reliable evaluation of the cathode interphase [66, 67, 68, 69]. As shown in Figure 3a, the initial discharge capacities at 0.1C were 180.5, 160.1, and 155.9 mAh g^−1^ for sNCM, sNCM@PI0.05, and sNCM@PI0.25, respectively. The marginally lower initial capacities of the coated samples can be attributed to transient Li^+^ diffusion limitations through the PI layer [49, 70], which are swiftly overcome upon electrochemical activation. As the current density increased, unmodified sNCM exhibited increasingly poor overlap of *dQ/dV* curves and pronounced polarization (Figure S10). While the capacity of unmodified sNCM sharply decreased to 81.7 mAh g^−1^ at 1C, the PI‐coated cathodes demonstrated superior capacities of 118.7 and 114.9 mAh g^−1^ for sNCM@PI0.05 and sNCM@PI0.25, respectively (Figure 3a). This enhanced rate performance is accompanied by improved *dQ/dV* curve overlap and attenuated polarization in coated electrodes (Figure S10), suggesting suppressed parasitic cathode‐electrolyte interface reactions and improved phase transition reversibility [71, 72]. In addition to interfacial stabilization, the suppression of Li^+^/Ni^2+^ cation mixing contributed to the reduced voltage polarization of the PI‐coated sNCM. Preventing Ni^2+^ from occupying the lithium layer effectively alleviated steric hindrance, thereby preserving continuous Li^+^ diffusion channels during cycling [27]. Notably, PI‐coated sNCM fully recovered its initial capacity upon returning from 2C to 0.1C, underscoring its structural resilience. In terms of long‐term cycling (Figure 3b), the sNCM@PI0.05 cathode delivered an initial discharge capacity of 125.2 mAh g^−1^ at 1C and exhibited a capacity retention of 83.6% after 400 cycles, outperforming both sNCM@PI0.25 (64.9%) and pristine sNCM (33.2%). The *dQ/dV* analysis (Figure 3c) revealed a substantially reduced voltage hysteresis (ΔE) in sNCM@PI0.05 (ΔE = 0.07 V) compared with the uncoated sNCM (ΔE = 0.24 V), underscoring enhanced phase transition reversibility (Figure S11). The superior cycling stability of sNCM@PI0.05 is also confirmed by its 85.4% capacity retention after 100 cycles at 0.5C (Figure 3d; Figure S12). Moreover, this advantage remains pronounced at an elevated rate of 2C (Figure S13), highlighting the robust protective function of the PI coating against interfacial degradation.

To evaluate the robustness of the PI coating under stringent electrochemical conditions, ASSBs using pristine sNCM and sNCM@PI0.05 were assembled and cycled at a high cut‐off voltage of 4.5 V vs Li/Li^+^. Although pristine sNCM exhibited slightly higher discharge capacities at low rates (0.1C and 0.2C), its performance deteriorated rapidly with increasing current density, retaining only 2.5% of its initial capacity at 2C (Figure 3e), accompanied by severe irreversible phase transitions and polarization (Figure S14). In contrast, sNCM@PI0.05 demonstrated improved cycling stability at high rates, retaining 15.2% of its initial capacity at 2C. As shown in Figure S15, sNCM@PI0.05 exhibited superior cycling stability at 1C compared to pristine sNCM, along with significantly reduced voltage polarization (0.03 V vs 0.07 V after 200 cycles). As summarized in Figure 3g, sNCM@PI0.05 exhibited a superior balance of cycling stability and rate capability compared to pristine sNCM. Remarkably, its cycling stability is highly competitive with state‐of‐the‐art polymer surface‐modified sNCM cathodes reported in the literature (Figure 3h, based on data in Table S2) [9, 18, 27, 30, 31, 32, 33, 34, 35, 36, 37, 38, 39]. These collective electrochemical results indicated that the PI coating was an effective interface‐engineering strategy for single‐crystal NCM cathodes in ASSBs. The optimal coating ratio (0.05 wt%) successfully balanced ionic transport and interfacial protection, whereas an excessive coating ratio (0.25 wt%) introduced detrimental barriers to ion transport, consistent with established principles in cathode coating design [26, 37, 38, 39].

Building upon the superior stability of sNCM@PI0.05 under standard operating conditions (4.3–4.5 V), we further explored the operational boundaries of this protective layer by extending the cutoff voltage to an extreme 4.8 V at 1C (Figure S16). Previous studies have established that polyimide frameworks possess exceptional intrinsic oxidative stability [4, 53, 54, 73, 74]. During prolonged cycling at 4.8 V, the bare sNCM exhibited steady degradation (49.5% capacity retention after 150 cycles). Meanwhile, the sNCM@PI0.05 experienced rapid capacity fading (retaining 14.4% after 123 cycles), whereas the sNCM@PI0.25 delivered an impressive capacity retention of 85.9% after 150 cycles. The contrasting behaviors of PI0.05 and PI0.25 can be attributed to a coupled chemo‐mechanical mechanism rather than the vulnerability of the PI itself. Specifically, the finite chemical scavenging sites of the 0.05 wt% coating might be chemically depleted by massive oxygen evolution at 4.8 V. Subsequently, the compromised thin polymer network is likely ruptured by catastrophic structural distortions of the cathode [75, 76, 77]. Conversely, the thicker polyimide layer in PI0.25 serves as a massive chemical buffer reservoir and provides robust mechanical confinement, withstanding the extreme electro‐chemo‐mechanical stress. These results suggest that while the 0.05 wt% coating represents the optimal balance for maximizing transport kinetics at 4.3 V, pushing the operational limit to 4.8 V requires a thicker PI layer to counteract severe oxidative and mechanical degradation.

### 2.3 Interfacial Evolution of Cathode/SSEs

**FIGURE 4:**
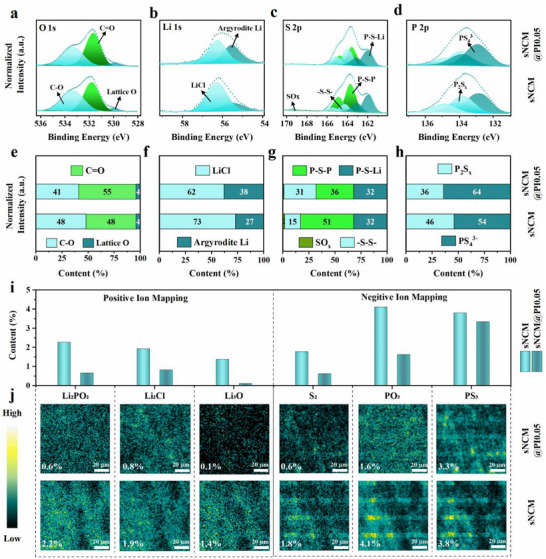
Ex situ XPS spectra of pristine sNCM and sNCM@PI0.05 cathodes after 100 cycles at 1C in the voltage range of 2.8–4.3 V vs Li/Li^+^: (a,e) O 1s, (b,f) Li 1s, (c,g) S 2p and (d,h) P 2p. (i) Normalized relative abundances (%) of positive and negative secondary ions detected by TOF‐SIMS. (j) TOF‐SIMS chemical species images from sNCM and sNCM@PI0.05 composite cathode after 100 cycles.

To unravel the underlying chemical and morphological evolution, *ex situ* XPS measurements were performed on cycled electrodes. As shown in Figure 4a,e, the O 1s spectra demonstrated a substantial suppression of interfacial side reactions, evidenced by a decrease in C‐O species from 48% in sNCM to 41% in sNCM@PI0.05 [57, 78]. Moreover, the uncoated sNCM exhibited a higher lattice oxygen BE (530.02 eV) than its PI‐coated counterpart (529.97 eV) after cycling, indicating considerable oxygen depletion at the surface of sNCM (Table S3) [79]. The robust interface established by the pre‐formed chemical bonding impeded direct contact between sNCM and solid electrolyte, which curtailed interface degradation pathways and improved cycling performance [12, 38]. The Li 1s spectra provide further evidence for suppressed parasitic reactions (Figure 4b,f). The lithium originating from the argyrodite electrolyte [80] increased from 27% for the pristine sNCM to 38% in the sNCM@PI0.05 composite. After 100 cycles, a peak emerged at 55.6 eV in the Li 1s spectra of both cathodes, which can be attributed to LiCl [81]. The LiCl species accounted for 73% in cycled sNCM, while the content of LiCl species was reduced to 62% in the cycled sNCM@PI0.05, highlighting the effectiveness of the PI interfacial layer in mitigating the decomposition of the electrolyte [82]. As indicated in Figure 4c,g, the dominant doublet at 161.2/162.3 eV corresponds to the PS_4_^3−^ moieties in LPSC lattice, while the other two components can be assigned to oxidized P‐S‐P species (162.9/164.2 eV) and ‐S‐S‐(163.5/164.6 eV), respectively [49, 70, 80]. Compared with sNCM, the integrated areas of these higher‐binding‐energy species (P‐S‐P, ‐S‐S‐) were significantly attenuated for sNCM@PI0.05. In addition, SO_x_‐related doublets at 164.4/168.5 eV were detected exclusively in sNCM, which were absent in sNCM@PI0.05. Meanwhile, the P_2_S_x_ signal decreased from 46% in pristine sNCM to 36% in sNCM@PI0.05 (Figure 4d,h). Collectively, these XPS observations demonstrated that the chemically anchored PI interface effectively inhibited electrolyte oxidation and mitigated parasitic side reactions.

High‐resolution time‐of‐flight secondary ion mass spectrometry (TOF‐SIMS) was further employed to probe the interfacial chemistry, with all ion signals normalized to total counts. As shown in Figure 4i,j, both cathodes revealed significant accumulation of negative‐ion species (PO_3_^−^, S^2−^ and PS_3_^−^ fragments) and positive‐ion species (Li_2_PO_2_^+^, Li_2_Cl^+^ and Li_3_O^+^) after 100 cycles, indicative of the irreversible structural transformation and surface side reactions [72, 82, 83]. However, these signals for the cycled sNCM@PI0.05 cathode were markedly reduced. For instance, the contents of Li_2_PO_2_^+^ and PO_3_^−^ decreased from 2.2% to 0.6% and from 4.1% to 1.6%, respectively. The consistency between TOF‐SIMS and XPS results collectively confirmed that the PI coating effectively mitigated parasitic interfacial reactions and irreversible phase transitions. TOF‐SIMS further resolved the chemical persistence and functionality of the polymer layer. Critically, characteristic PI fragments (e.g., NH_4_^+^, C_2_H_6_N^+^, C_3_H_8_N^+^, C_5_H_5_^+^, C_5_H_7_^+^, C_5_H_9_^+^, C_6_H_5_^+^, C_6_H_6_^+^, C_6_H_9_^+^, C_6_H_7_^+^, C_7_H_7_^+^, C_7_H_9_^+^) were detected exclusively on the coated surfaces of sNCM@PI0.05, demonstrating the structural persistence of the coating during extended cycling (Figure S17). The detection of oxygen‐containing cations (CH_3_NO_2_^+^, CH_2_NO^+^, C_5_H_5_O^+^) exclusively at PI‐modified interfaces possibly supported a chemisorptive scavenging mechanism. The pre‐formed chemical bonding empowers the PI layer to capture and immobilize reactive oxygen, thereby inhibiting their migration and the consequent degradation of the solid electrolyte.

### 2.4 Characterization of Cathode Structural Evolution

**FIGURE 5:**
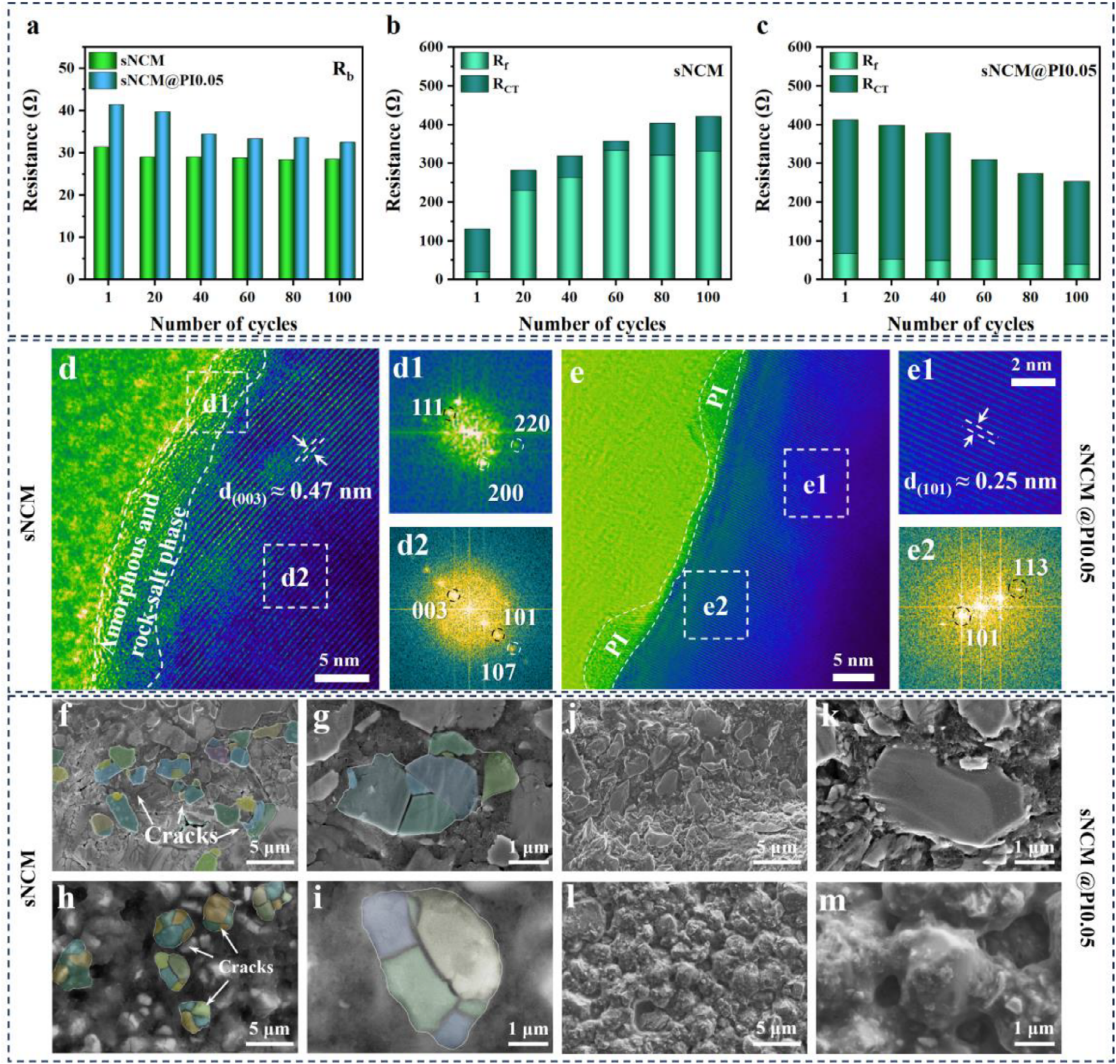
(a–c) Evolution of interfacial resistances for pristine sNCM and sNCM@PI0.05 cathodes, extracted from Nyquist plots at the first, 20th, 40th, 60th, 80th, and 100th cycle (Table S4). HR‐TEM images of cycled cathodes after 100 cycles at 1C: (d) sNCM (images d1 and d2 corresponding to position d1 and d2 in (d,e) sNCM@PI0.05 (images e1 and e2 corresponding to position e1 and e2 in (e). SEM images of sNCM cathode composite (f–i) and sNCM@PI0.05 cathode composite (j–m) after 100 cycles at a rate of 1C.

Electrochemical impedance spectroscopy (EIS) provided critical insights into the dynamic interfacial evolution enabled by the PI coating. As shown in Figure 5a–c, the Nyquist plots were fitted using an equivalent circuit (Figure S18) in which the high‐frequency intercept corresponds to the bulk resistance of the solid electrolyte within the composite electrode (R_b_), the mid‐frequency semicircle represents the cathode–solid electrolyte interfacial resistance (R_f_​), and the low‐frequency arc reflects the charge transfer resistance (R_CT_) [32, 84]. Initially, the R_b_ for sNCM@PI0.05 (41.5 Ω) was higher than that of sNCM (31.5 Ω), consistent with the low ionic conductivity of the as‐formed PI layer. During cycling, R_b_ remained relatively stable for both electrodes (decreasing slightly to 28.6 Ω for sNCM and 32.6 Ω for sNCM@PI0.05), indicating improved interfacial contact. At the first cycle, the R_f_ and R_CT_ for sNCM@PI0.05 were 68.5 Ω and 345.1 Ω, respectively, significantly higher than those of sNCM (21.2 Ω and 111.4 Ω). However, over 100 cycles, pristine sNCM exhibited a 15.8‐fold increase in R_f_ (from 21.2 to 332.5 Ω). This impedance growth can be attributed to severe interfacial side reactions and progressive contact loss due to microcrack propagation [13, 43], which is corroborated by subsequent SEM analyses. In contrast, PI‐coated sNCM demonstrated a remarkable 41% reduction in R*_f_* ​ (from 68.5 to 40.2 Ω). Concurrently, R_CT_​ decreased by 38% (from 345.1 to 214.6 Ω) for the coated cathode, whereas it slightly declined (from 111.4 to 89.6 Ω) for the pristine sNCM. The concurrent reduction in both Rf and RCT suggested the existence of a unique dynamic adaptation mechanism at the PI‐modified interface during electrochemical cycling, which progressively enhances Li^+^ transport [84, 85, 86]. Consequently, the total interfacial impedance (R_f​_ + R_CT_) of sNCM@PI0.05 decreased by 38% over extended cycling, a rare phenomenon that highlights the power of reactive polymer‐based interface engineering in stabilizing high‐energy solid‐state cathodes.

To deconvolute the overlapping impedance responses, distribution of relaxation times (DRT) analysis was performed (Figure S19) [87, 88]. Based on established timescale domains for sulfide‐based batteries, the resolved peaks are assigned to bulk solid electrolyte (R_b_, 10^−6^ to 10^−5^ s), interfacial contact (R_f_, 10^−5^ to 10^−2^ s), and charge‐transfer (R_CT_, 10^−2^ to 10^0^ s) resistances [89, 90, 91]. For the pristine sNCM, the R_f_ splits into two distinct peaks: R_f1_ (chemical contact impedance) and R_f2_ (mechanical contact degradation) [90]. During 100 cycles, the R_f2_ undergoes an obvious increase from 30.9 to 104.3 Ω. Concurrently, the R_CT_ increases from 13.6 to 44.7 Ω (Table S5). This explicit decoupling indicated that the degradation of bare sNCM is dominated by continuous chemo‐mechanical interface degradation and the severe loss of active charge‐transfer sites. Conversely, sNCM@PI0.05 maintains a single, unified R_f_ peak, demonstrating that the viscoelastic polymer coating effectively bridges interfacial gaps and suppresses mechanical contact loss. Despite a slightly higher initial resistance from the pristine PI barrier, subsequent cycling triggers an adaptive activation. Consequently, R_f_ and R_CT_ steadily decrease and stabilize at 26.8 and 107.3 Ω after 100 cycles, quantitatively confirming that the PI layer functions as a robust chemo‐mechanical buffer for long‐term interfacial stability.

The critical role of the PI coating in mitigating cathode degradation was further evidenced by HR‐TEM. After 100 cycles, the pristine layered structure of sNCM underwent extensive phase transformation into spinel and rock‐salt phases, resulting in an ∼4 nm thick interfacial layer (Figure 5d). In contrast, PI‐coated sNCM maintained a well‐ordered layered structure with clearly resolved (101) lattice fringes near the surface (Figure 5e), evidencing the efficiency of the PI coating in suppressing phase transformation. As shown in Figure 1a, preserving high cationic ordering intrinsically stabilizes the layered lattice framework, helping to prevent its irreversible phase transition into a resistive rock‐salt phase [39, 51]. This intrinsic structural retention beneficially contributes to the observed enhancements in long‐term cycling stability. Complementary cross‐sectional SEM revealed stark differences in microstructural evolution between the two cathodes. As shown in Figure 5f,g, the sNCM exhibited pronounced intergranular cracking after 100 cycles (Figure S20). The sNCM@PI0.05 cathode retained its original microstructure and particle morphology with minimal cracking (Figure 5j,k; Figure S21). This phenomenon can be attributed to the viscoelastic PI interphase that dynamically accommodated the anisotropic volume changes [92]. The PI coating (Young's modulus ∼7.16 GPa vs. pristine ∼9.76 GPa) acts as a mechanical buffer, distributing stress uniformly across the particle surface and preventing localized stress concentration, initiating crack propagation in rigid cathode materials. This stress‐dissipating capability was further corroborated by top‐view SEM analysis. After the first cycle, both cathode materials exhibited structurally intact particles with intimate electrode‐electrolyte interfacial contact (Figure S22). After 100 cycles, pristine sNCM exhibited severe intragranular cracking and fragmentation (Figure 5h,i; Figure S23), whereas sNCM@PI0.05 retained remarkably intact particles with minimal cracking (Figure 5l,m; Figure S24).

The synergistic interplay between electrochemical activation and mechanical adaptation fosters a dynamically self‐optimizing cathode–electrolyte interphase, which evolves favorably during cycling. This interfacial evolution manifests as a continuous reduction in total impedance, while the viscoelastic PI layer effectively accommodates anisotropic volume changes in sNCM particles, thereby preserving mechanical integrity and interfacial contact. As to the coating non‐uniformity in Figure 5e after cycling, it may stem primarily from electrochemically induced structural reorganization of the PI interphase. It is worth noting that this nanoscale heterogeneity does not imply a complete loss of coating integrity. As shown in Figure S25, the characteristic elements of the PI coating (F and N) are observed to be continuously distributed across various regions of the cathode surface, suggesting that its overall conformal encapsulation and functionality are largely preserved after cycling. Furthermore, the restricted penetration of P, S, and Cl into the bulk of sNCM indicates the sustained protective functionality of the PI layer. Consequently, the localized thickness variations observed at the nanoscale can be interpreted as a dynamic adaptive reconstruction, which possibly arises from the coupled effects of stress‐induced polymer redistribution and interfacial by‐product incorporation [93]. During repeated (de)lithiation, anisotropic volume changes in sNCM generate localized interfacial stresses. PI's viscoelastic nature could accommodate these strains through polymer chain rearrangement, causing localized thinning or thickening at stress‐concentrated regions [1, 18, 94, 95]. In addition, LPSC decomposition products become embedded within the PI matrix during cycling, modifying local TEM contrast and morphology. This self‐optimizing interfacial paradigm transcends conventional interface engineering approaches, representing a fundamental advancement toward durable, high‐performance all‐solid‐state batteries.

## 3 Conclusion

This work demonstrates a transformative interfacial engineering strategy for Ni‐rich cathodes in sulfide‐based ASSBs through the molecularly precise construction of a PI coating. The PI interphase concurrently addresses the coupled challenges of interfacial chemical degradation and mechanical contact loss via synergistic mechanisms. Robust carboxylate–transition metal coordination effectively removes residual lithium species and suppresses oxygen release, while the viscoelastic compliance of the PI layer dynamically accommodates anisotropic volume changes during cycling. Furthermore, the electrochemically driven self‐optimization of the reactive polymer interface continuously reduces interfacial impedance, resulting in exceptional cycling stability. The optimized sNCM@PI_0.05_ cathode achieves 83.6% capacity retention after 400 cycles at 1C under a 4.3 V cutoff voltage and 90.2% after 200 cycles at a high cut‐off voltage of 4.5 V. Collectively, this chemically and mechanically adaptive interfacial design establishes a scalable and generalizable strategy for developing durable, high‐energy all‐solid‐state batteries, overcoming the intrinsic limitations of conventional rigid or inert coating systems.

## 4 Experimental Section

### 4.1 Materials

All reagents were analytical grade and used without further treatment. sNCM, LPSC, lithium metal disks, indium foil, and Super‐P carbon were purchased from Shenzhen Kejing Material Technology Co., Ltd. (Shenzhen, China). Anhydrous N‐methylpyrrolidone (NMP), BAPB, 6FDA, and isoquinoline were purchased from Energy Chemical (Shanghai, China).

### 4.2 Synthesis of PAA

PAA was synthesized via solution polycondensation of 6FDA and BAPB in anhydrous NMP under an argon atmosphere, using isoquinoline as a catalyst. The molar ratio of 6FDA to BAPB was maintained at 1:1. In a typical procedure, BAPB (0.542 g, 1.22 mmol) was first dissolved in 5 mL of NMP in a three‐neck flask under continuous stirring at room temperature. Subsequently, 6FDA (0.450 g, 1.22 mmol) was added to the diamine solution, followed by the addition of 75 µL of isoquinoline. The resulting mixture was stirred for 24 h to yield a homogeneous 20.0 wt.% PAA solution.

### 4.3 Preparation of sNCM@PI Cathodes

The 20.0 wt.% PAA solution was diluted with NMP to yield a 1.0 wt.% PAA solution. Subsequently, 500 mg of pristine sNCM powder was ultrasonically dispersed in 5 mL of anhydrous NMP in a three‐neck round‐bottom flask under vigorous magnetic stirring (1500 rpm) for 1 h. Then, 25 µL or 50 µL of the 1.0 wt.% PAA solution—corresponding to sNCM@PI0.05 and sNCM@PI0.25, respectively—and 75 µL of isoquinoline were sequentially added to the suspension. The resulting mixture was stirred for 1 h to ensure homogeneity. The composite suspension was then subjected to a stepwise thermal imidization process under a nitrogen atmosphere to facilitate PI formation (Figure S1). The temperature was incrementally raised as follows: 80°C (1 h) → 100°C (1 h) → 120°C (1 h) → 140°C (1 h) → 160°C (1 h) → 180°C (1 h) → 200°C (1 h). Following the final imidization step, the reaction mixture was held at 200°C under a gentle N_2_ purge for approximately 2 h to ensure complete removal of residual solvent and volatile byproducts. The resulting solid product was collected and further dried under vacuum at 150°C for 3 h to yield the PI‐coated sNCM cathode composite.

### 4.4 Material Characterizations

TGA was performed on a TGA‐Q50 instrument under a nitrogen atmosphere at a heating rate of 10°C min^−1^. FT‐IR spectra were recorded using a Thermo Scientific Nicolet 6700 spectrometer. XRD patterns were collected on a Rigaku SmartLab diffractometer using Cu *Kα* radiation (λ = 1.5406 Å) over a 2θ range of 10–90° at a scan rate of 10° min^−1^, with operating conditions of 40 kV and 40 mA. Morphological and microstructural characteristics were examined using a Hitachi SU‐70 SEM equipped with an energy‐dispersive X‐ray spectroscopy system. AFM (Bruker Dimension Icon) was operated in tapping mode using a TESP‐V2 silicon tip (resonance frequency: 320 kHz; spring constant: 40 N m^−1^). The Young's modulus was evaluated using instrumented nanoindentation (iMicro Nanoindenter, KLA‐Tencor) equipped with a diamond Berkovich indenter. The elastic modulus was derived from the initial unloading stiffness of the load‐displacement curves following the Oliver‐Pharr methodology. To ensure statistical reliability, a minimum of four independent indentations were performed on each sample. For cross‐sectional SEM, electrodes were harvested in a discharged state in an argon‐filled glovebox and polished using an argon ion beam milling system (JEOL IB‐19520CCP). HR‐TEM (JEOL F200) was conducted at 200 kV. XPS was performed on a Thermo Scientific ESCALAB 250Xi system using a monochromatic Al *Kα* X‐ray source (1486.6 eV).

### 4.5 Fabrication of ASSBs

The composite cathode was prepared by blending sNCM (or sNCM@PI), LPSC, and Super P in a weight ratio of 70:27:3 using an agate mortar for manual milling for approximately 15 min. The anode consisted of a Li–In alloy sandwiched between two 10 mm indium foils and a 6 mm lithium foil. First, 100 mg of LPSC powder was cold‐pressed at 150 MPa to form a dense electrolyte pellet (10 mm diameter). Then, 6–8 mg of the cathode composite was added onto one side and pressed at 300 MPa, followed by pressing the anode side at 150 MPa.

### 4.6 Electrochemical Measurements

The electrochemical performance of the *ASSBs* was evaluated using a NEWARE CT4008‐T battery cycler. Galvanostatic charge–discharge tests were conducted in the voltage range of 2.2–3.7 V and 2.2–3.9 V vs Li–In (equivalent to 2.8–4.3 V and 2.8–4.5 V vs Li/Li^+^) at current densities of 0.1C, 0.2C, 0.5C, 1C, and 2C (1C = 200 mA g^−1^). Galvanostatic charge–discharge tests were conducted in the voltage range of 2.2–4.2 V vs Li–In (equivalent to 2.8–4.8 V vs Li/Li^+^) at current densities of 1C (1C = 200 mA g^−1^). EIS was performed over a frequency range of 1 Hz–1 MHz with a 10 mV AC amplitude at 0.1C. DRT analysis based on Tikhonov regularization was employed to deconvolute the overlapping interfacial impedance. Both real and imaginary EIS data were utilized, discarding high‐frequency inductive artifacts. Discretization was performed using Gaussian radial basis functions (FWHM coefficient = 0.5) and second‐order derivative regularization. A globally fixed regularization parameter (λ = 0.005) was strictly applied to all computations. TOF‐SIMS was carried out on an IONTOF GmbH instrument equipped with a 30 keV Bi_3_^+^ primary ion gun, achieving a lateral resolution of 100 nm. Samples were transferred under argon, and a 100 × 100 µm^2^ area was analyzed with a primary ion dose below 1.66 × 10^14^ ions cm^−2^ to ensure static SIMS conditions.

## Author Contributions

Jiatao Wu: Investigation, Conceptualization, Data Curation, Formal analysis, Writing – original draft, Writing – review & editing. Wenjin Li: Methodology, Resources, Writing – review & editing. Rui Wang: Methodology. Peng Wang: Investigation. Kaiyuan Deng: Validation. Chengshuai Chang: Formal analysis, Resources. Chuan Xie: Resources. Lei Yao: Supervision. Guangliang Gary Liu: Resource, Supervision, Writing – review & editing.

## Conflicts of Interest

The authors declare no conflicts of interest.

## Supporting information




**Supporting File**: advs75595‐sup‐0001‐SuppMat.docx.

## Data Availability

The data that support the findings of this study are available from the corresponding author upon reasonable request.
