# Supplementary material for: Mechanically Adaptive Polyimide Interfaces for Stable High‐Voltage NCM–Sulfide All‐Solid‐State Batteries
Source: Adv Sci (Weinh). 2026 May 8;13(43):e75595. doi: 10.1002/advs.75595 (PMC13335770; doi:10.1002/advs.75595)
Supplement: Supplementary file 1 — Supporting File: advs75595‐sup‐0001‐SuppMat.docx. [file ADVS-13-e75595-s001.docx]

Supporting Information

**Mechanically Adaptive Polyimide Interfaces for Stable High-Voltage NCM–Sulfide All-Solid-State Batteries**

*Jiatao Wu*^a^*^#^, Wenjin Li*^a^*^#^, Rui Wang*^a^*, Peng Wang*^b^*, Cheng Liu*^a^*, Kaiyuan Deng*^a^*, Chengshuai Chang*^a^*, Chuan Xie*^a^*, Lei Yao*^a^*, Guangliang Gary Liu*^a^***

^a^Guangdong Provincial Key Laboratory of New Energy Materials Service Safety, College of Materials Science and Engineering, Shenzhen University, Shenzhen, 518060, China

^b^Faculty of Civil Aviation and Aeronautics, Kunming University of Science and Technology, Kunming 650500, China

*Corresponding author: Guangliang Gary Liu, e-mail: ggliu@szu.edu.cn


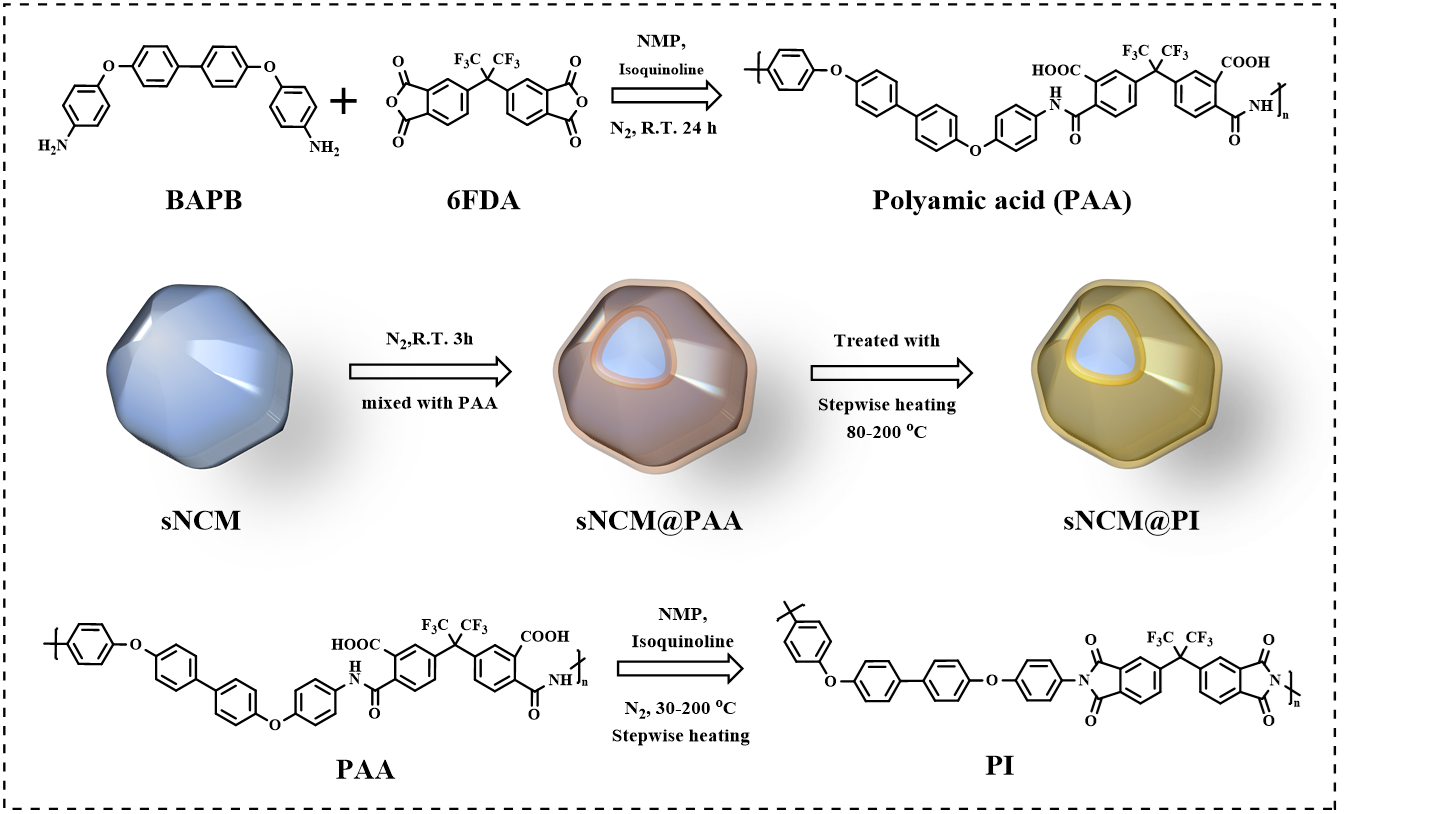


Figure S1. Schematic illustration of the fabrication process for polyimide-coated single-crystal NCM811 (sNCM@PI). The procedure involves: (i) synthesis of poly (amic acid) (PAA) *via* polycondensation of 6FDA and BAPB; (ii) mixing PAA with sNCM particles to enable uniform adsorption; and (iii) stepwise thermal imidization (30→ 80 → 120 →140 →160 →180→200 °C, 1 h per step) to convert the adsorbed PAA into a conformal 6FDA–BAPB polyimide (PI) coating.

**

**

**Figure S2.** Gel permeation chromatography trace of the synthesized PI. The measurement was carried out in tetrahydrofuran at 35 °C with a flow rate of 1.0 mL min⁻¹, using a refractive index detector. Molecular weights were determined relative to linear polystyrene standards. The obtained number-average molecular weight (*Mₙ*), weight-average molecular weight (*M_w_*), and polydispersity index (PDI = *M_w_*/*M_n_*) are 51 kDa, 79 kDa, and 1.56, respectively.





**Figure S3.** Thermogravimetric analysis (TGA) curves of pristine sNCM, sNCM@PI0.05, and sNCM@PI0.25 under N₂ atmosphere. The measurements were performed from 30 to 800 °C at a heating rate of 10 °C min^-1^.

TGA was performed under a nitrogen atmosphere (10 °C min⁻¹, 30–800 °C) to estimate the polyimide content and evaluate thermal behavior. Prior to analysis, all samples were pre-dried at 100 °C for 10 min under N₂ to reduce physisorbed moisture. The TGA profiles exhibit three mass-loss regions common to all samples. The first, occurring between room temperature and ~130 °C, is assigned to the desorption of physically adsorbed water and residual solvents. ^1^ The second stage (130–300 °C) likely arises from the dehydration of surface lithium hydroxide hydrate (LiOH·H₂O) and/or decomposition of loosely bound lithium residues. ^1^ Critically, thermal degradation of the 6FDA-PI matrix commences near 300 °C, as established in prior literature. ^2^ Thus, the mass loss observed between 300–800 °C may reflect: (i) decomposition or carbonization of a thin polyimide layer, and (ii) thermal decomposition of surface lithium species—particularly Li₂CO₃, which begins to decompose above ~700 °C under inert atmospheres,^3^. In the 300–800 °C interval, the mass losses were 0.44% for pristine sNCM, 0.57% for sNCM@PI0.05 , and 0.75% for sNCM@PI0.25 . The incremental losses (0.13% and 0.31%) roughly correspond to the nominal polyimide loadings (0.05 wt% and 0.25 wt%), suggesting that the coating process is reproducible and scalable. However, complete volatilization of 6FDA-BAPB is highly unlikely. Therefore, partial carbonization during high-temperature pyrolysis is more plausible. Moreover, the additional mass loss observed in the coated samples may also stem from lattice oxygen release from the Ni-rich sNCM at elevated temperatures.^3a^ The 0.44% loss for pristine sNCM is consistent with trace surface lithium residues (e.g., Li₂CO₃, LiOH), commonly observed in Ni-rich cathodes.^3^

**
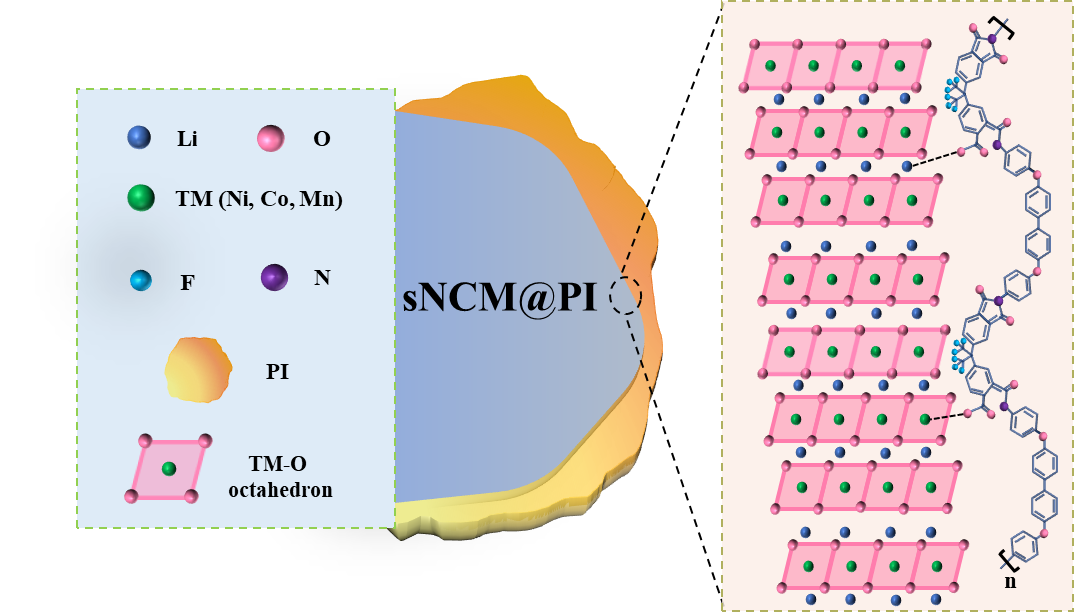
**

**Figure S4**. Proposed interfacial interaction mechanism between the PAA precursor and the sNCM surface. Carboxylic acid (–COOH) groups in PAA may coordinate with surface-exposed Ni²⁺/Ni³⁺ ions or associate with residual Li⁺ species (e.g., from Li₂CO₃ or LiOH), forming chemical linkages that promote uniform adsorption prior to thermal imidization.^4^ Although the final polyimide (PI) coating is chemically inert, the PAA precursor can anchor to the cathode surface through carboxylate–metal interactions, facilitating conformal coverage-a mechanism supported by analogous polymer–inorganic systems.^5^


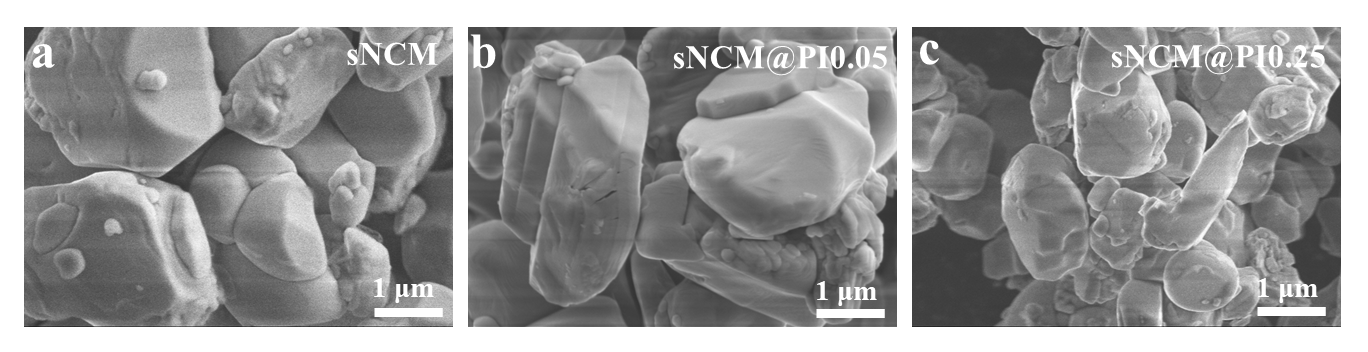


**Figure S5.** Scanning electron microscopy (SEM) images of (a) sNCM, (b) sNCM@PI0.05 and (c) sNCM@PI0.05.


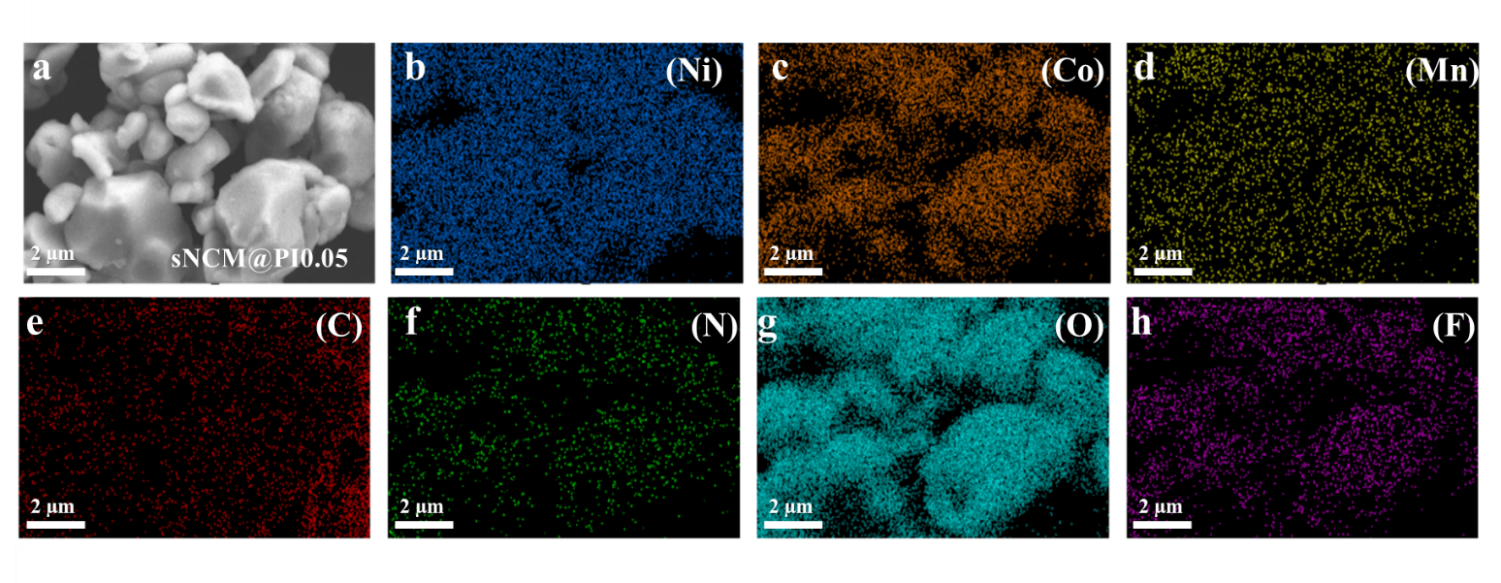


**Figure S6.** SEM images (a) of sNCM@PI0.05 with corresponding EDS elemental mappings of (b-h) Ni, Co, Mn, C, N, O, F, respectively.


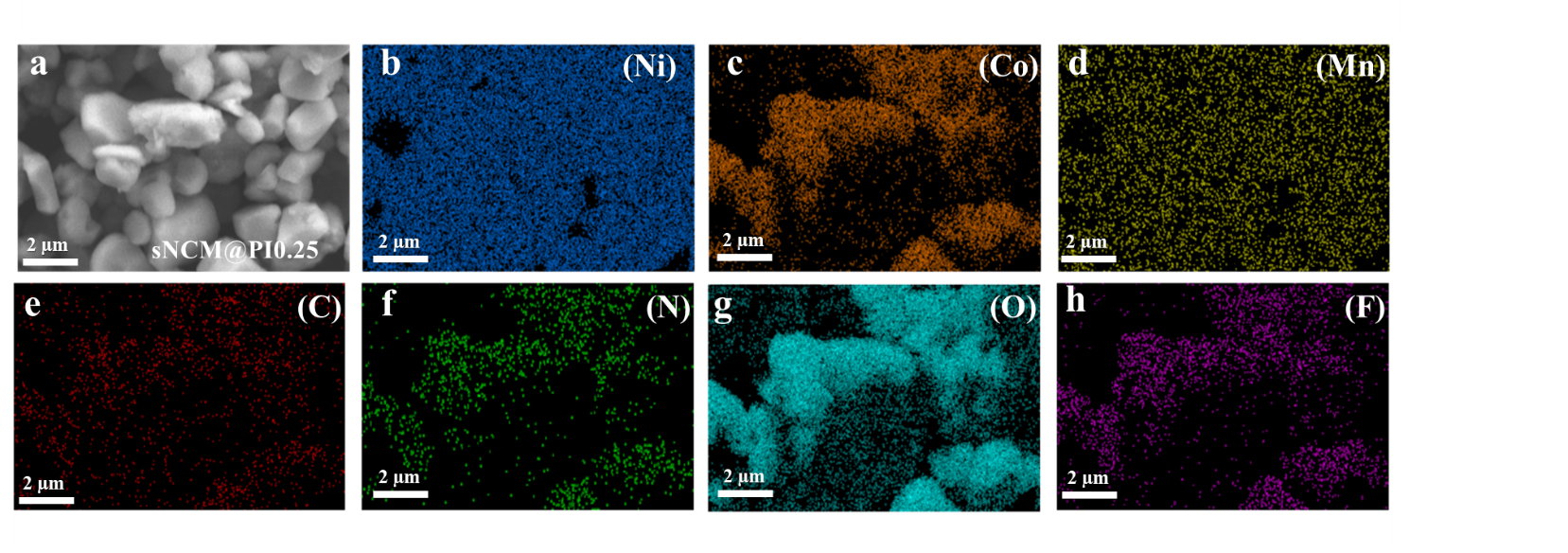


**Figure S7.** SEM images (a) of sNCM@PI0.25 with corresponding EDS elemental mappings of (b-h) Ni, Co, Mn, C, N, O, F, respectively.


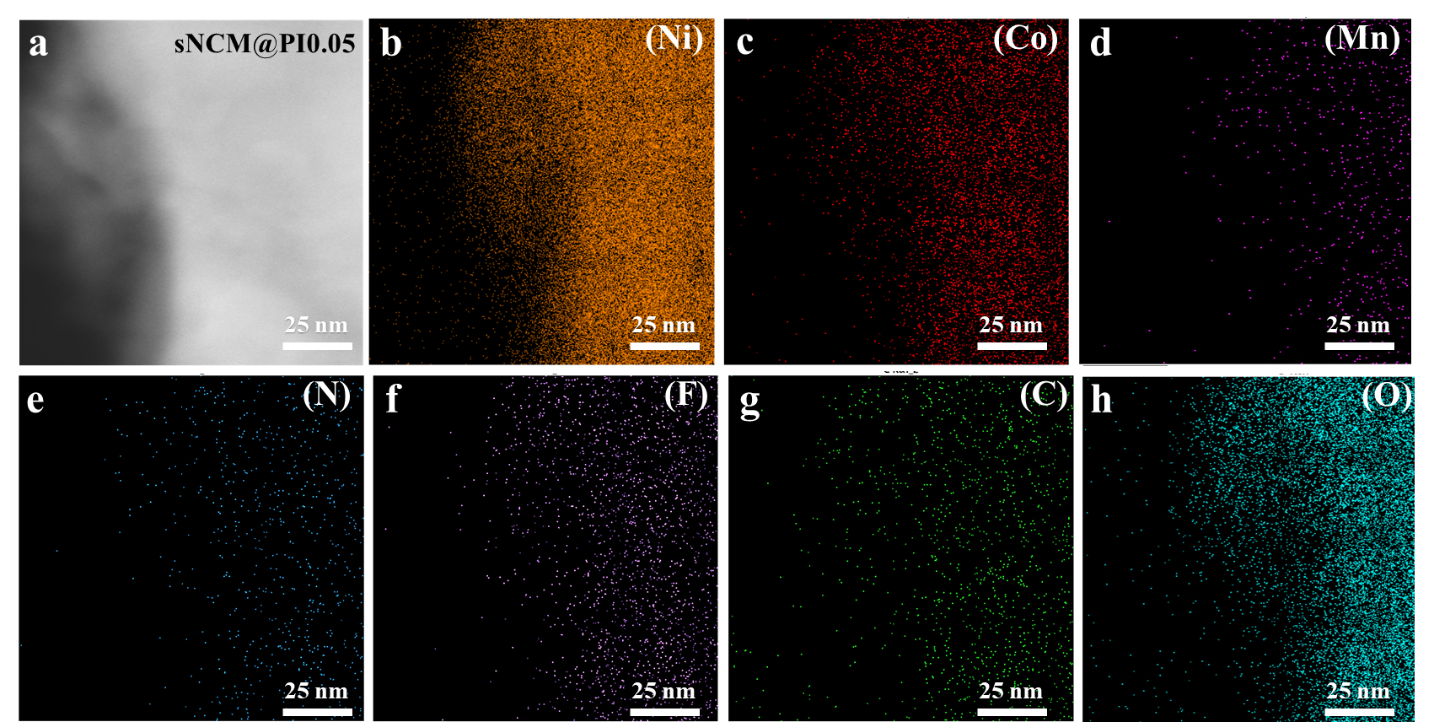


**Figure S8.** High-resolution transmission electron microscopy (HR-TEM) images (a) of sNCM@PI0.05 with corresponding EDS elemental mappings of (b-h) Ni, Co, Mn, N, F, C, O, respectively.





**Figure S9.** Representative load-displacement curves for the pressed composite cathode pellets of (a) sNCM811 and (b) sNCM@PI0.05, obtained by nanoindentation.

**
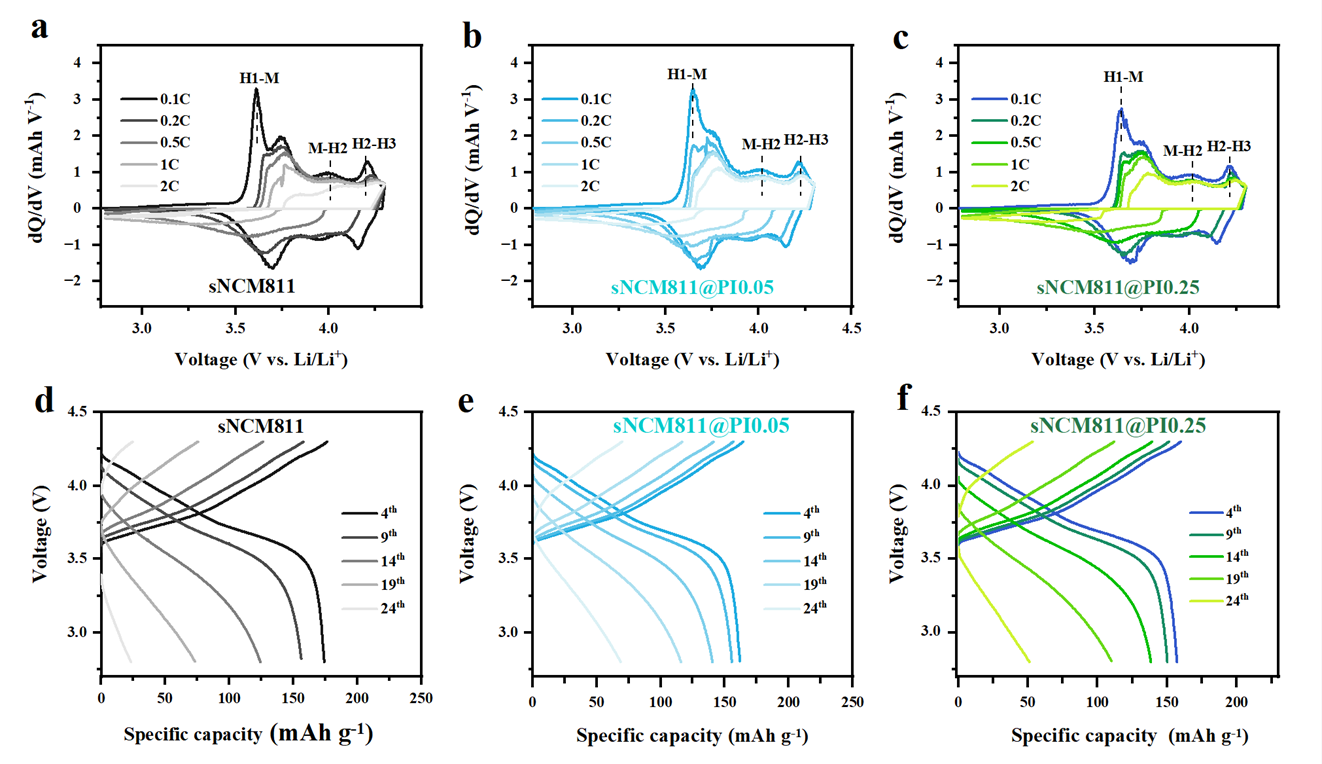
**

**Figure S10.** Differential capacity (*dQ/dV*) profiles of (a) sNCM, (b) sNCM@PI0.05 and (c) sNCM@PI0.25 at various current rates. (d-f) Galvanostatic charge-discharge profiles of (d) sNCM, (e) sNCM@PI0.05 and (f) sNCM@PI0.25 at selected cycles. All measurements were conducted within a voltage range of 2.8−4.3 V vs. Li/Li⁺.


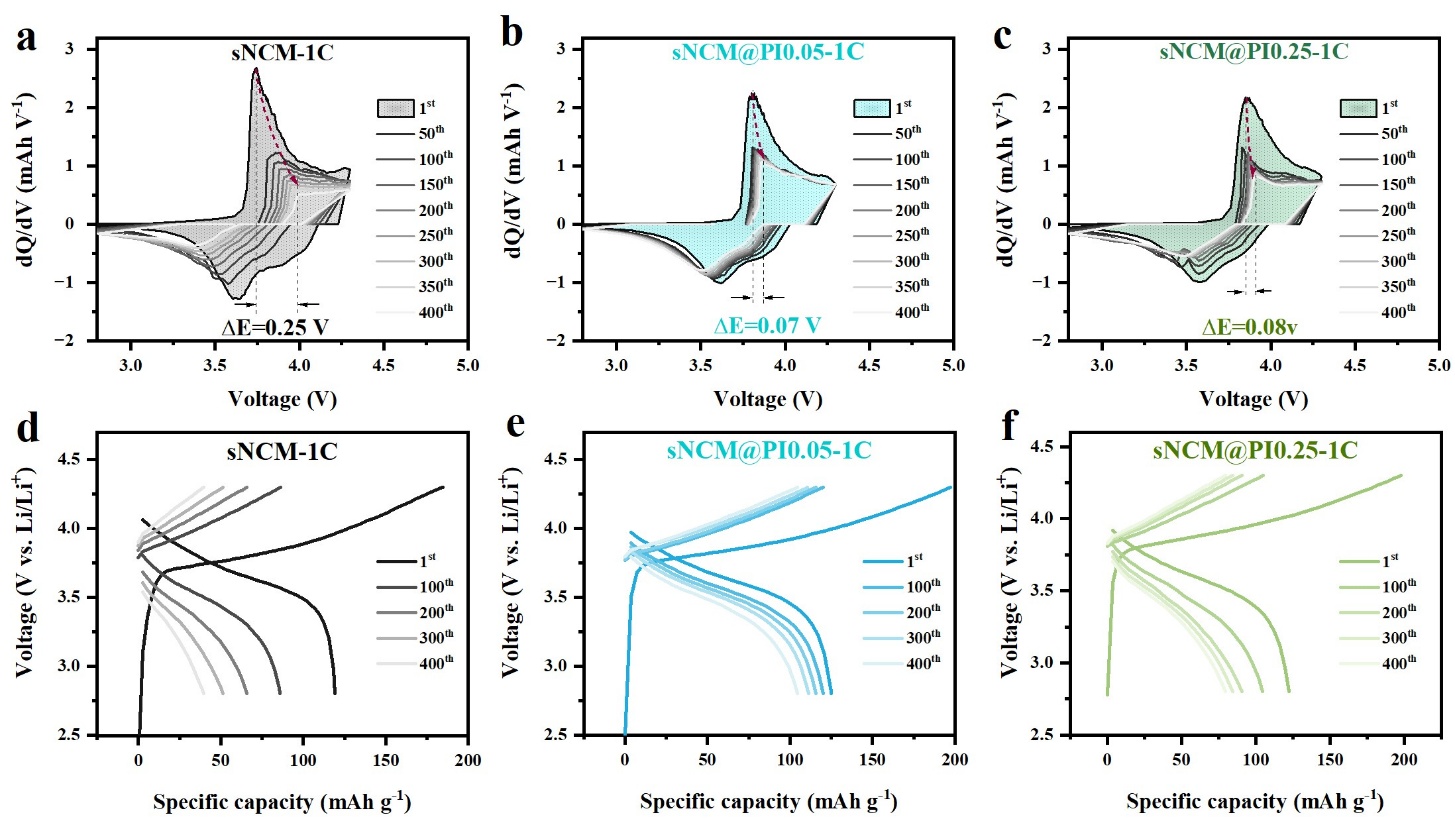


**Figure S11.** The *dQ/dV* curves of (a) sNCM, (b) sNCM@PI0.05, and (c) sNCM@PI0.25 at selected cycles. Corresponding galvanostatic charge-discharge profiles of (d) sNCM, (e) sNCM@PI0.05 and (f) sNCM@PI0.25 at 1 C. All tests were performed within a voltage window of 2.8−4.3 V vs. Li/Li⁺.


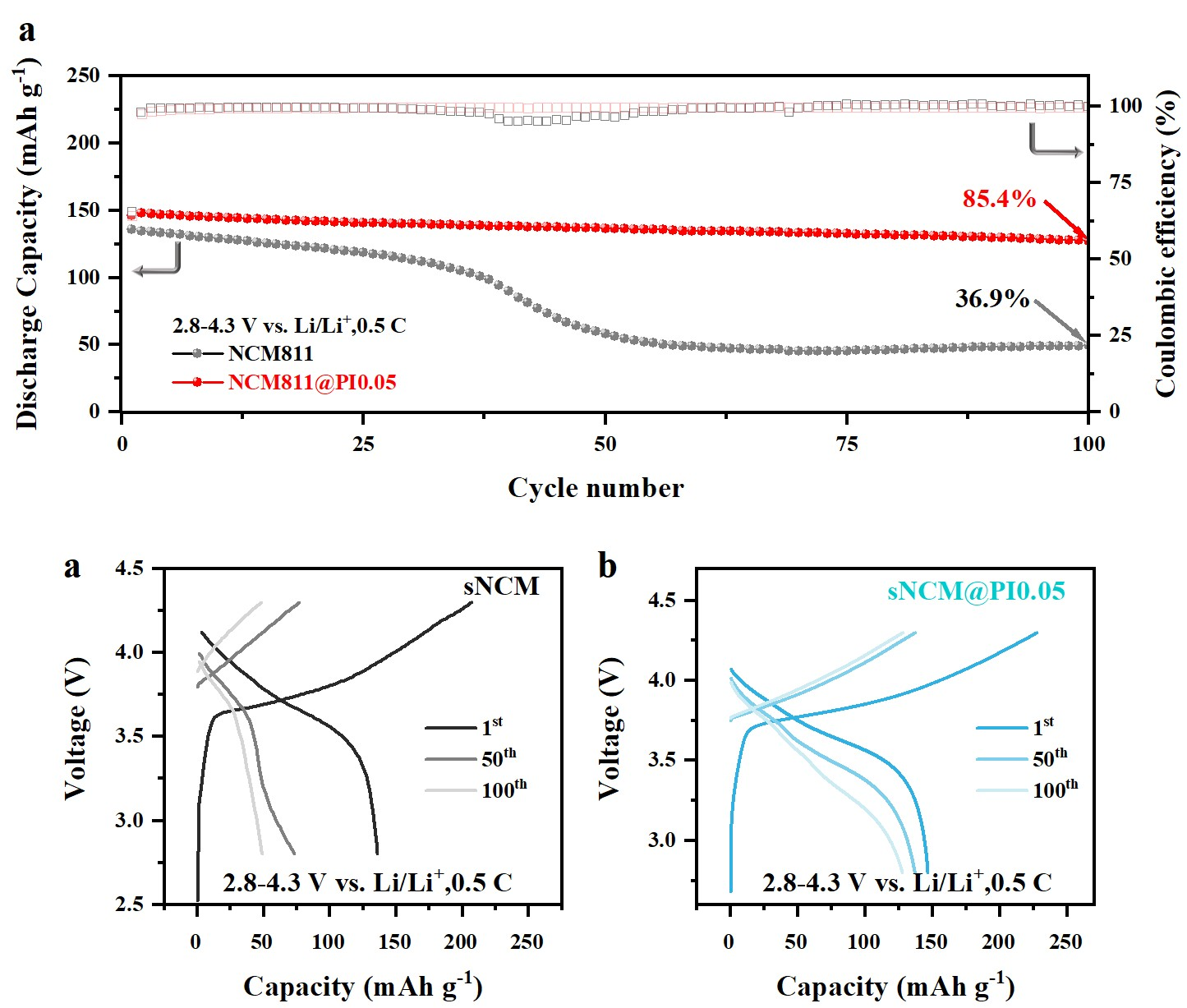


**Figure S12.**  Galvanostatic charge-discharge profiles at selected cycles for (a) sNCM and (b) sNCM@PI0.05, measured at 0.5 C within a voltage range of 2.8−4.3 V vs. Li/Li⁺ at 25 °C.





**Figure S13.** (a) Long-term cycling performance of sNCM and sNCM@PI0.05 at 2 C. Galvanostatic charge-discharge profiles at selected cycles for (b) sNCM and (c) sNCM@PI0.05. All measurements were carried out within a voltage range of 2.8−4.3 V vs. Li/Li⁺ at 25 °C.


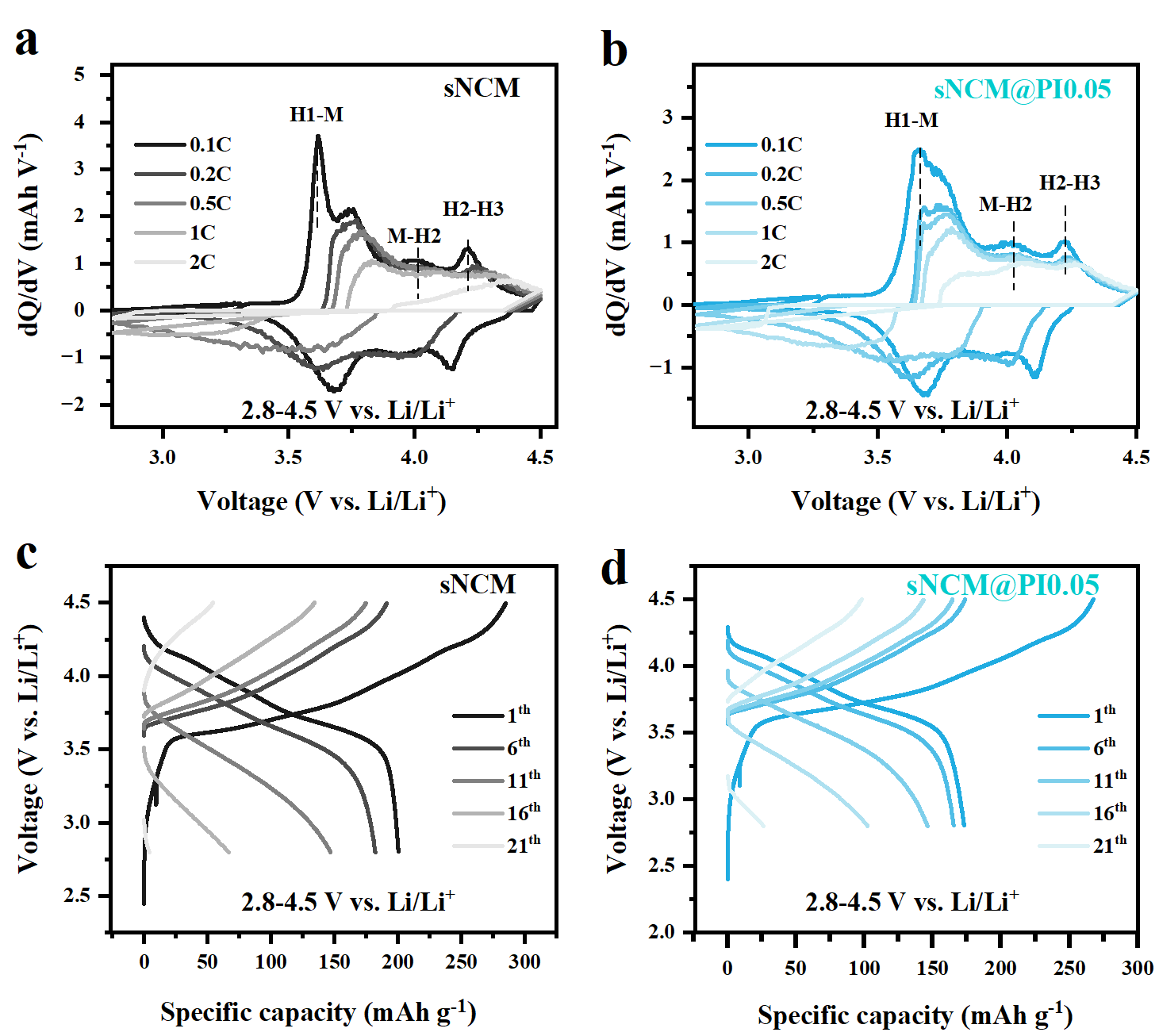


**Figure S14.** Electrochemical performance at a high cut-off voltage of 4.5 V vs. Li/Li⁺.  The *dQ/dV* curves at various rates for (a) sNCM and (b) sNCM@PI0.05. Galvanostatic charge-discharge profiles at selected cycles for (c) sNCM and (d) sNCM@PI0.05.


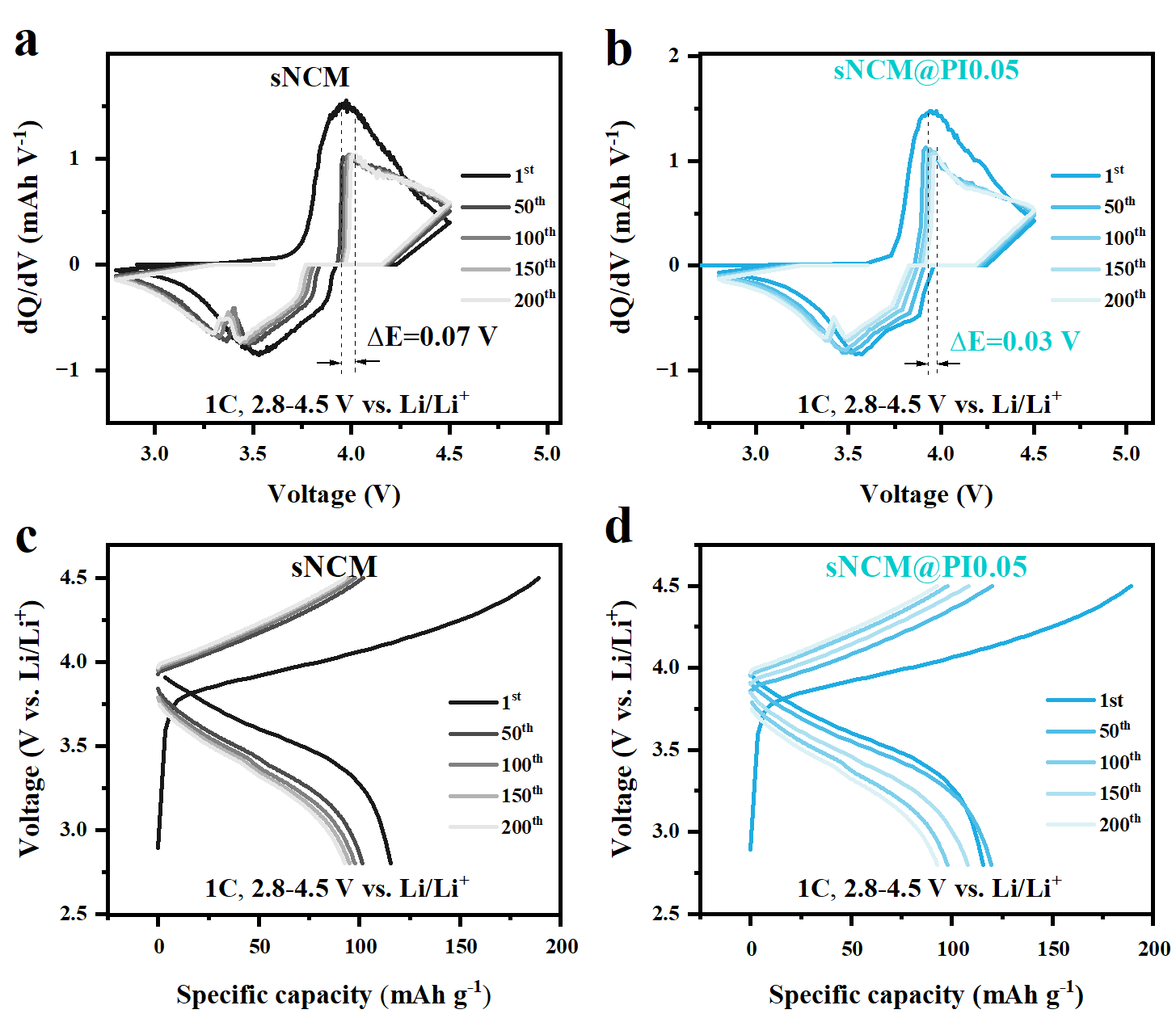


**Figure S15.** Electrochemical performance at a high cut-off voltage of 4.5 V vs. Li/Li⁺ and 1 C rate. *dQ/dV* curves at selected cycles for (a) sNCM and (b) sNCM@PI0.05. Galvanostatic charge-discharge profiles at selected cycles for (c) sNCM and (d) sNCM@PI0.05.

**

**

**Figure S16.** (a) Long-term cycling performance of sNCM, sNCM@PI0.05 and sNCM@PI0.25 at 1 C. Galvanostatic charge-discharge profiles at selected cycles for (b) sNCM, (c) sNCM@PI0.05 and (d)sNCM@PI0.25. All measurements were carried out within a voltage range of 2.8−4.8 V vs. Li/Li⁺ at 25 °C.


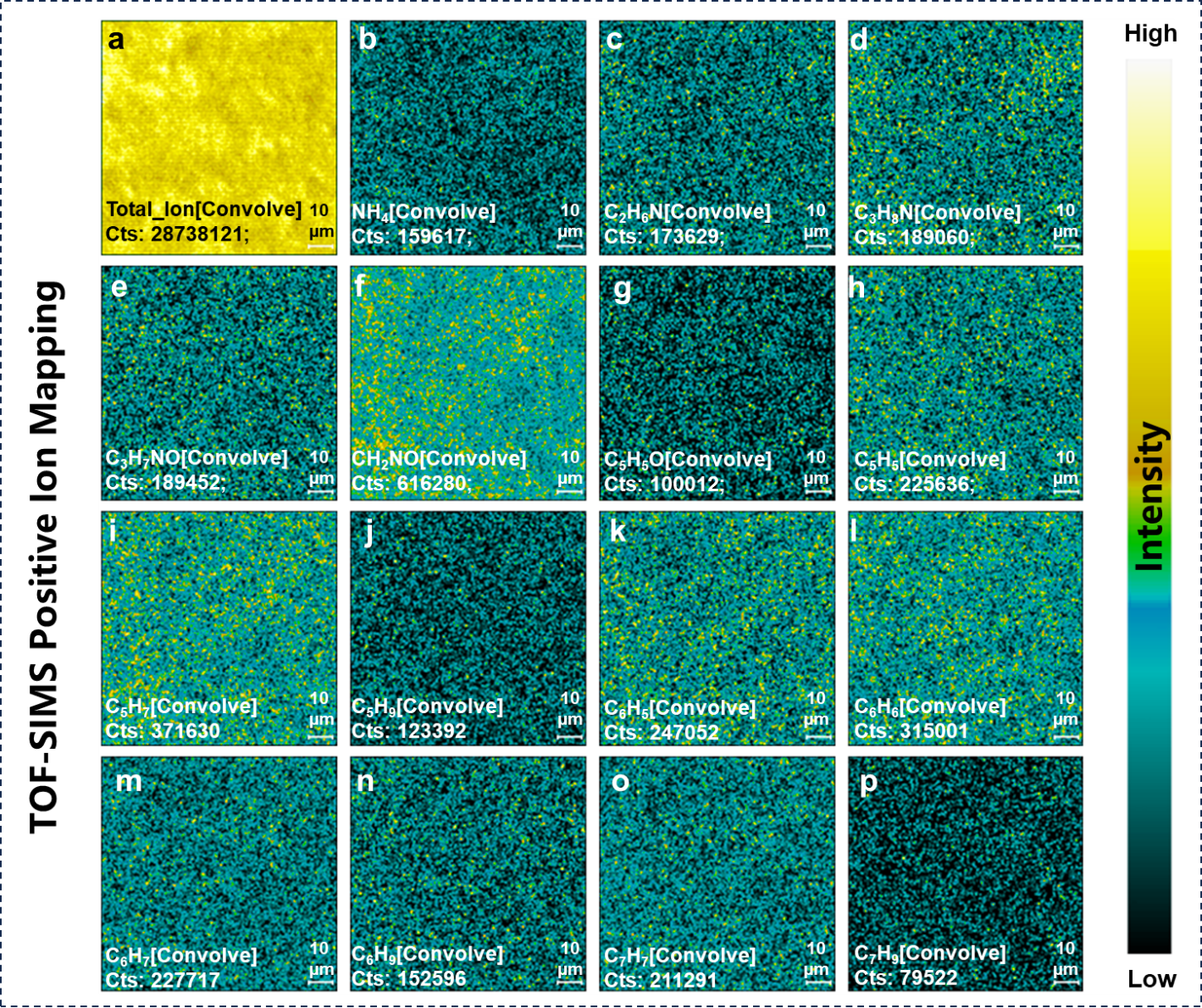


**Figure S17.** High-resolution time-of-flight secondary ion mass spectrometry (TOF-SIMS) chemical species images from sNCM@PI0.05 composite cathode after 100 cycles at 1 C in the voltage range of 2.8–4.3 V vs. Li/Li^+^.


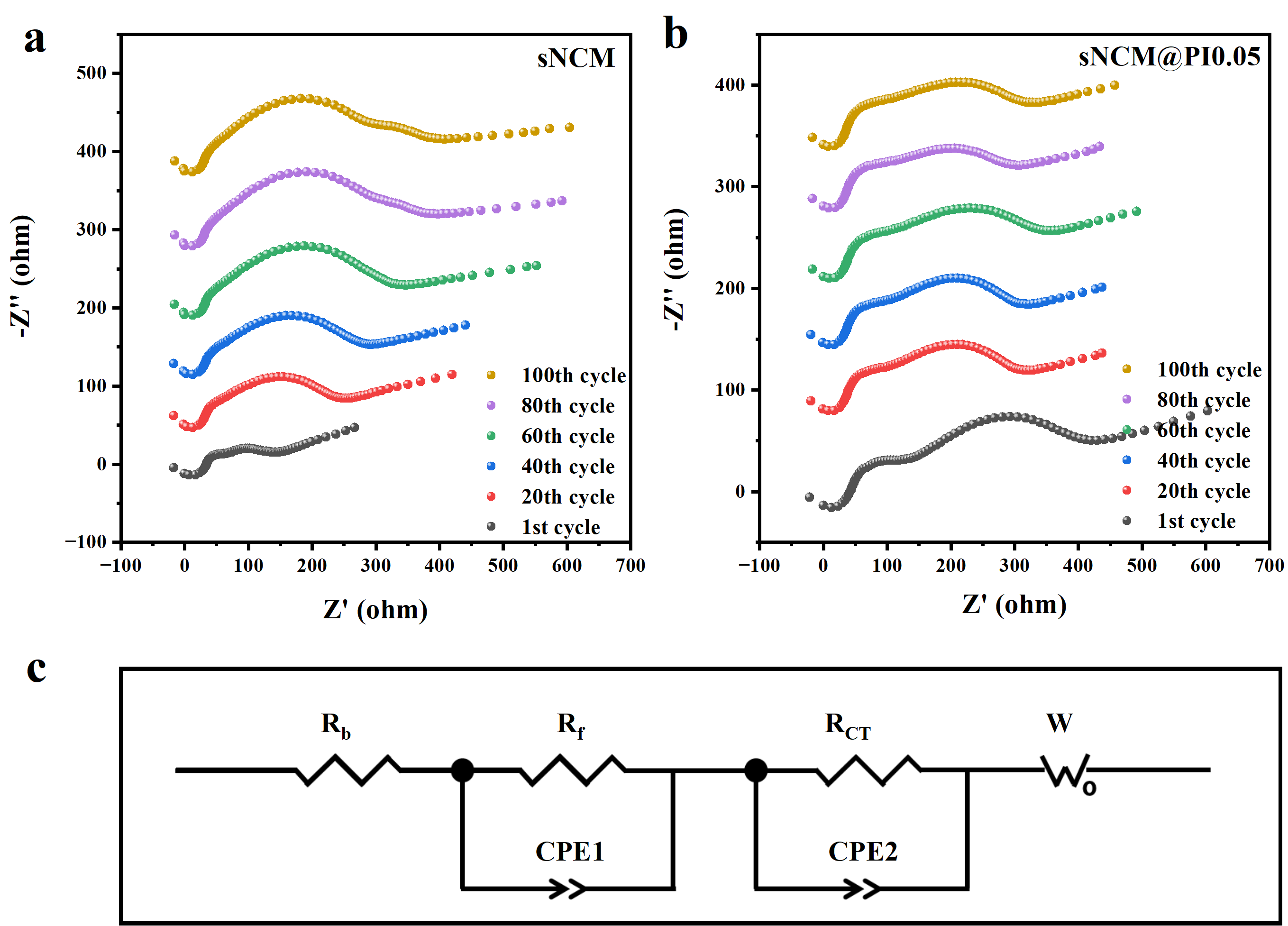


**Figure S18** Nyquist plots of (a) sNCM and (b) sNCM@PI0.05 at the 1st, 20th, 40th, 60th, 80th, and 100th cycle. (c) Electrochemical impedance spectroscopy (EIS) fitting equivalent circuit for sNCM and sNCM@PI0.05 cells.





**Figure S19.** Evolution of interfacial kinetics decoupled by Distribution of Relaxation Times (DRT) analysis. DRT profiles of the (a) bare sNCM and (c) sNCM@PI0.05 cathodes during prolonged cycling (from the 1st to the 100th cycle). The colored background regions denote the distinct timescale domains corresponding to specific electrochemical processes: the bulk solid electrolyte resistance (R_b_), the interfacial contact resistance (R_f,_ which splits into R_f1_ and R_f2_ for bare sNCM), and the charge-transfer resistance (R_CT_). Quantitative resistance evolution extracted *via* DRT peak integration for the (b) bare sNCM and (d) sNCM@PI0.05 cathodes at the 1st, 20th, 40th, 60th, 80th, and 100th cycle.


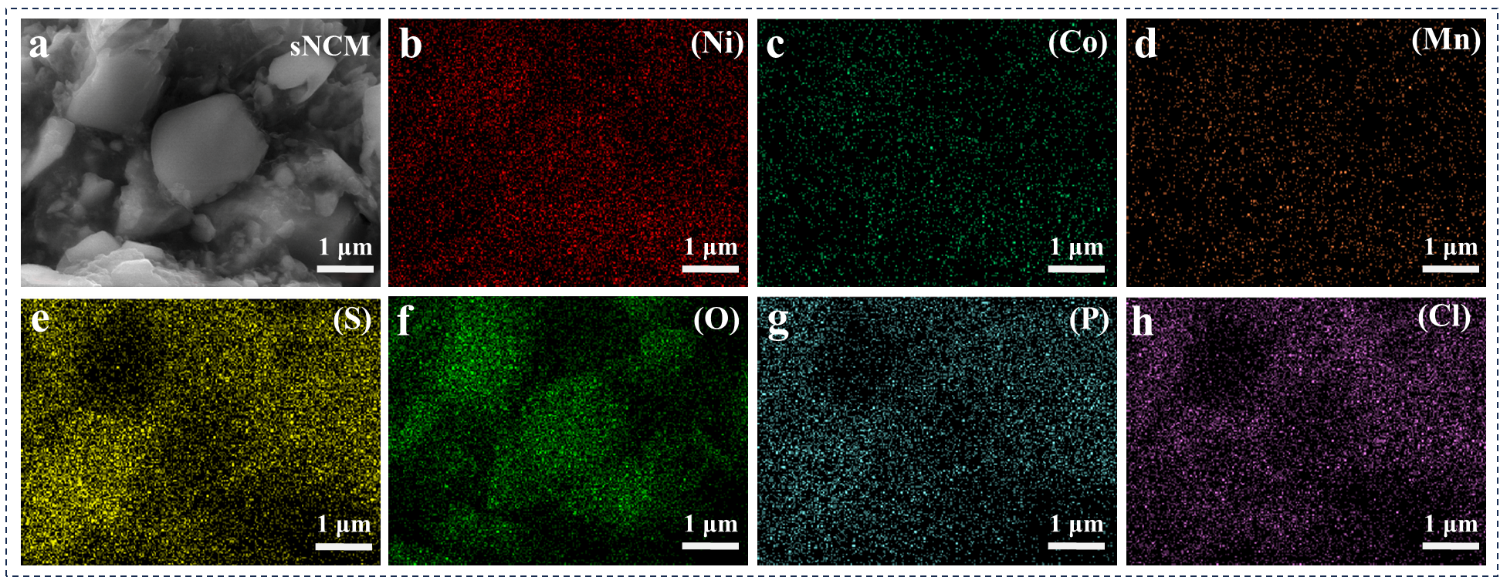


**Figure S20.** Cross-sectional Energy-dispersive spectroscopy (EDS) elemental maps of the sNCM cathode composite after 400 cycles, depicting the distribution of Ni, Mn, Co, S, O, P and Cl.


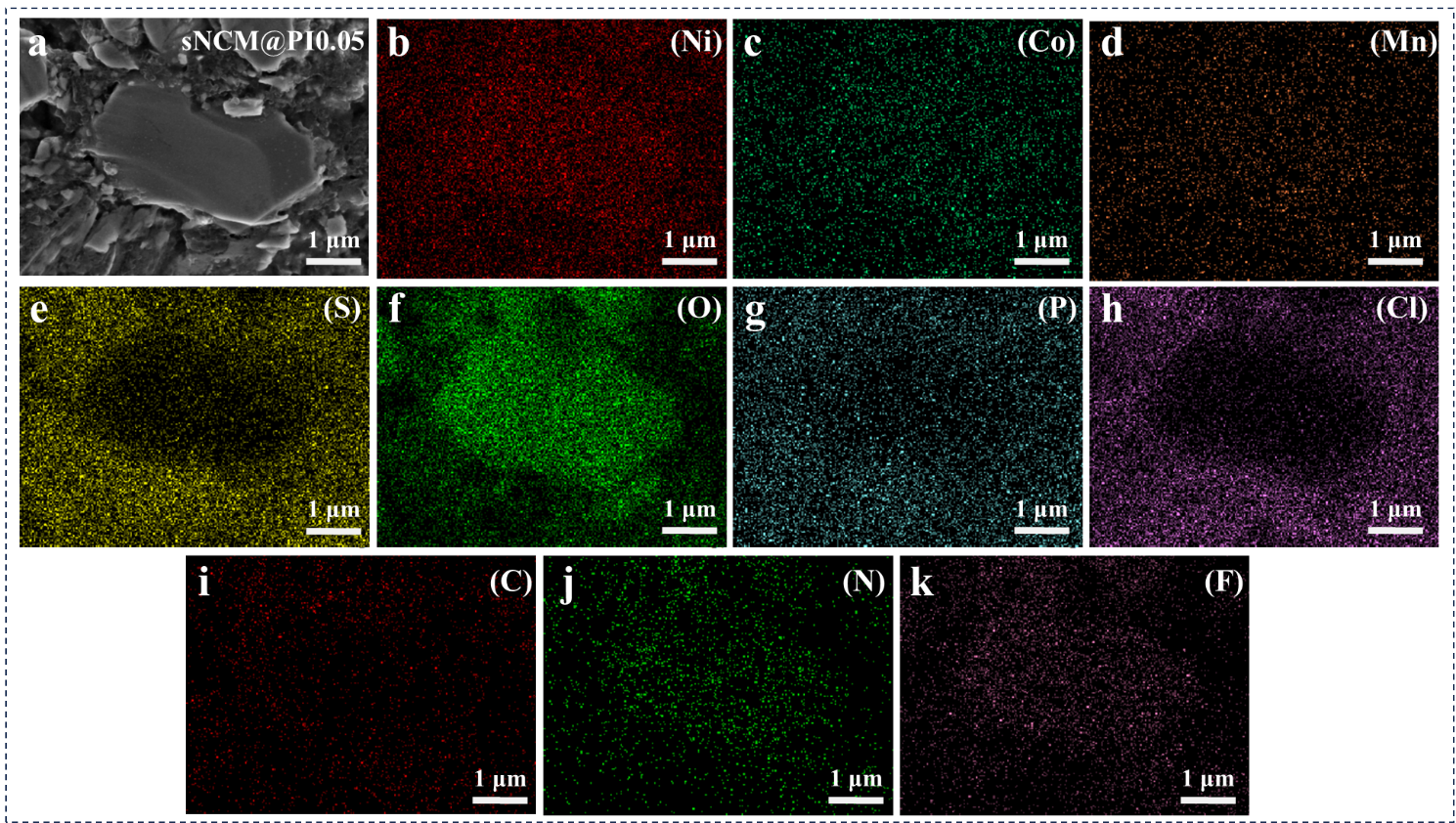


**Figure S21.** Cross-sectional EDS elemental maps of the sNCM@PI0.05 cathode composite after 400 cycles, depicting the distribution of Ni, Co, Mn, S, O, P, Cl, C, N and F.


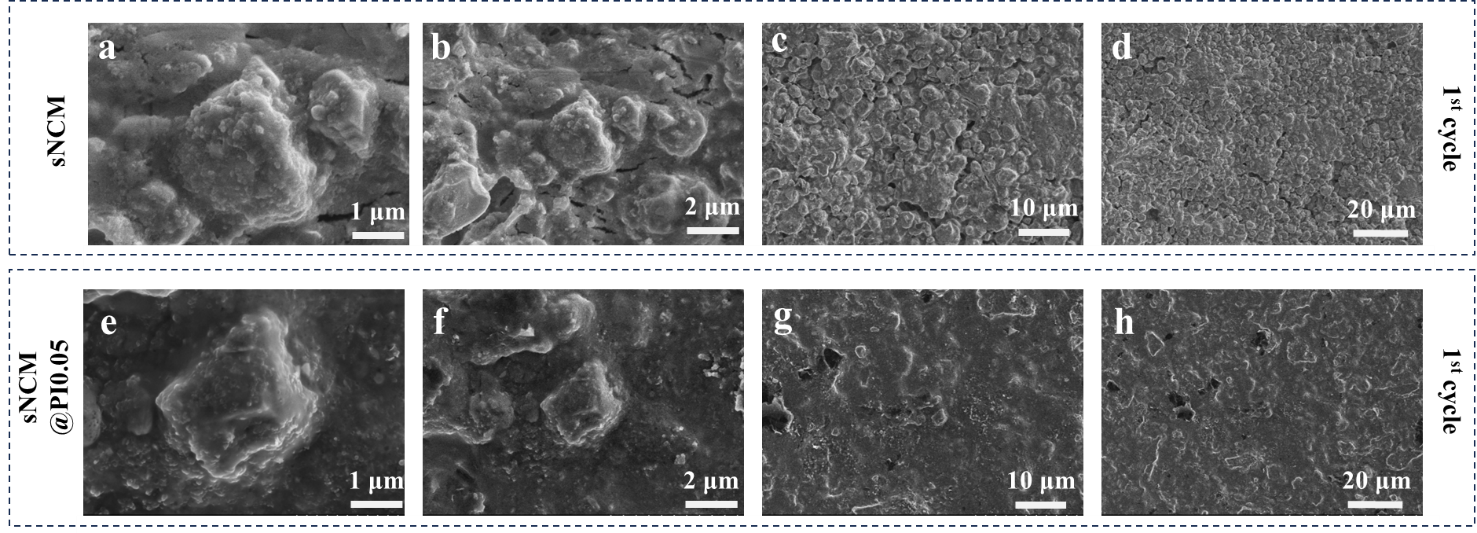


**Figure S22.** Top-view SEM images of sNCM (a-d) and sNCM@PI0.05 (e-h) cathode composites after first cycle.


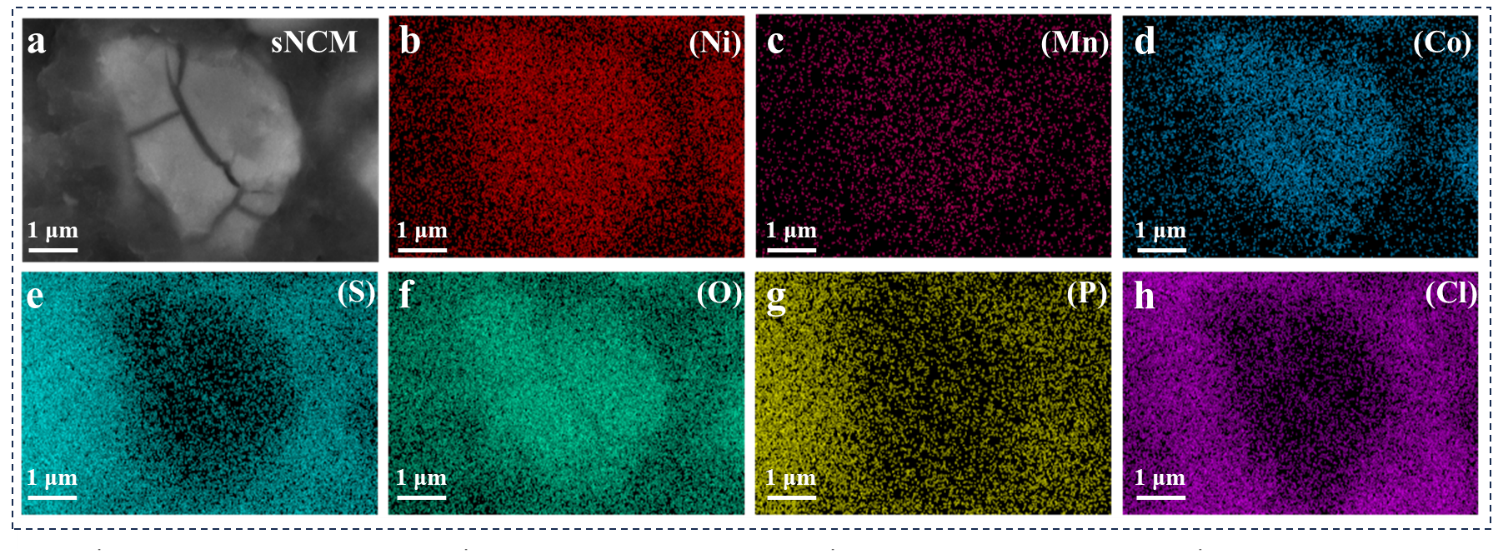


**Figure S23.** Top-view EDS elemental maps of the sNCM cathode composite after 400 cycles, depicting the distribution of Ni, Co, Mn, O, P, S, and Cl.


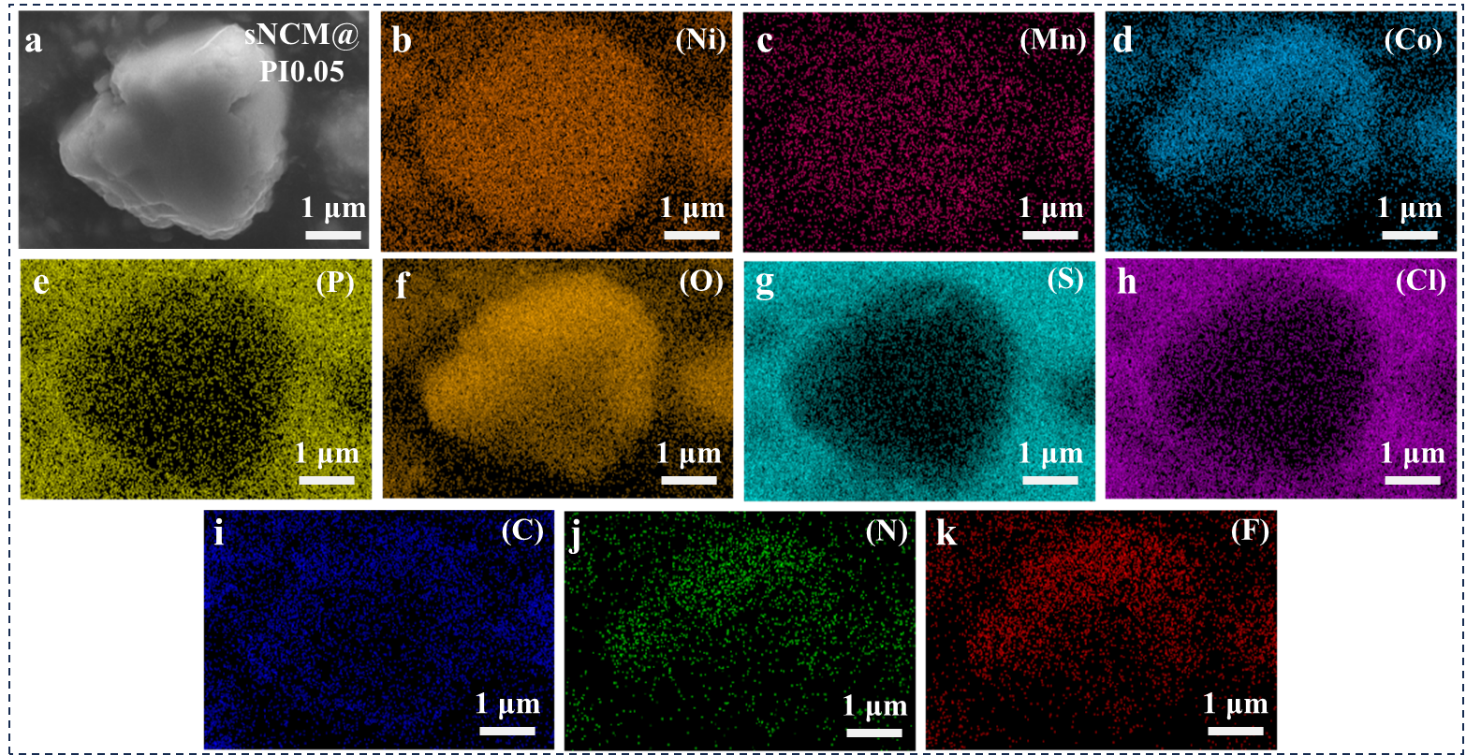


**Figure S24.** Top-view EDS elemental maps of the sNCM@PI0.05 cathode composite after 400 cycles, depicting the distribution of Ni, Co, Mn, P, O, S, Cl, C, N, and F.


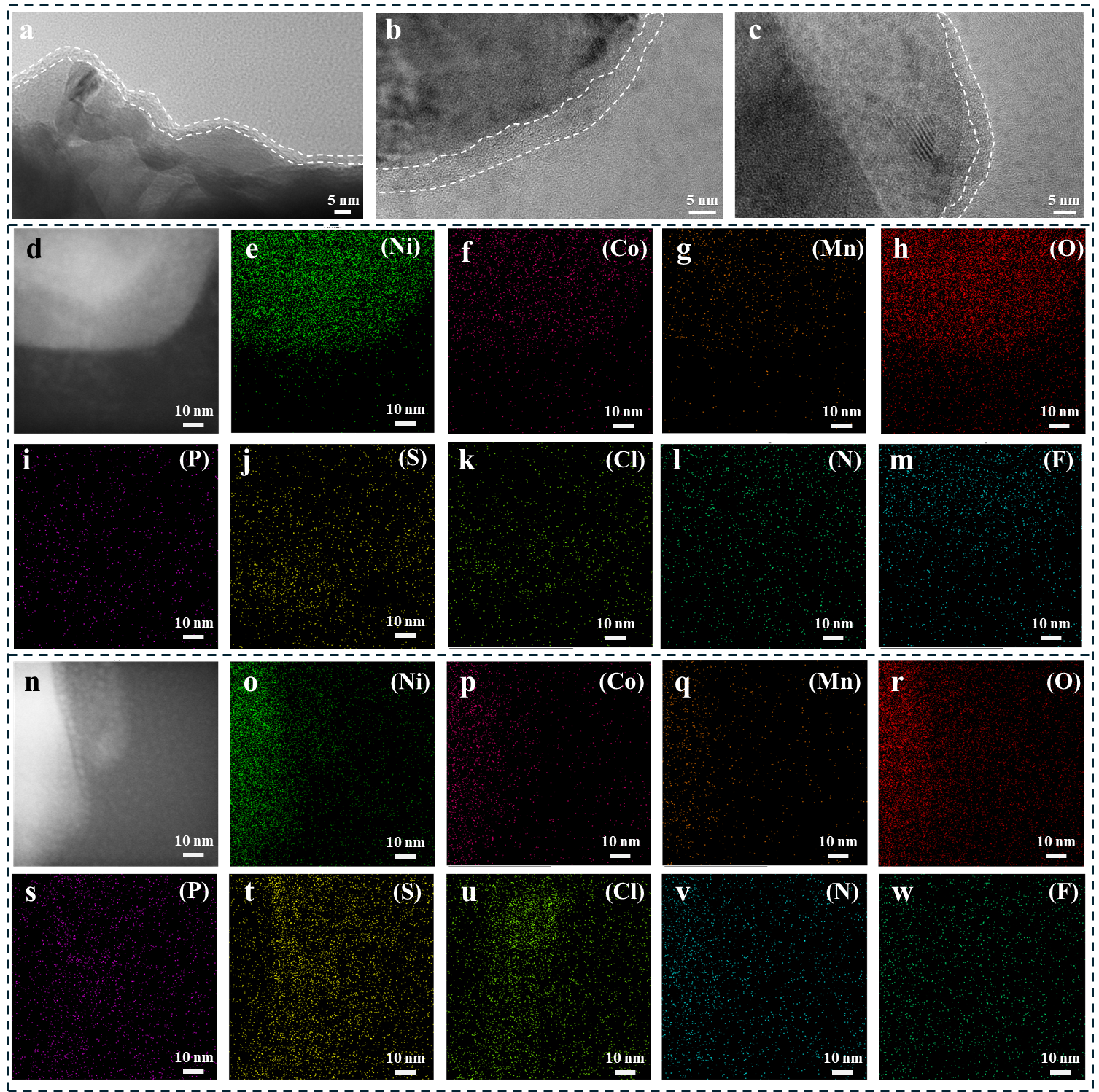


**Figure S25.** Representative post-cycling HR-TEM images and EDS elemental mappings of sNCM@PI0.05 cathode after 100 cycles at 1C. (a–c) HR-TEM images from three different regions. (d–m) EDS mappings of Ni, Co, Mn, O, P, S, Cl, N, and F for region (b). (n–w) EDS mappings of Ni, Co, Mn, O, P, S, Cl, N, and F for region (c).

| **Table S1. XPS binding energies of key functional groups identified from peak fitting analysis for the sNCM and sNCM@PI0.05 cathodes.** | | | | | |
| --- | --- | --- | --- | --- | --- |
| **Species** | | **Assignment** | **sNCM^a^**  **(eV)** | **sNCM@PI0.05**  **(eV)** | [**sNCM@PI0.2**](mailto:NCM811@P3HT0.2)**5**  **(eV)** |
| **C 1s** | C=O | | 288.55 | 289.55 | 289.36 |
|  | C-O | | 286.21 | 287.18 | 286.01 |
|  | C-C | | 284.8 | 284.8 | 284.8 |
| **O 1s** | C-O | | 533.50 | - - | - - |
|  | C=O | | 531.98 | 531.66 | 531.55 |
|  | Lattice O | | 529.53 | 529.24 | 528.95 |
| **Ni 2p** | Ni^2+^ | | 855.33 | 855.06 | 855.11 |
|  | Ni^3+^ | | 856.54 | 856.53 | 855.56 |
|  | Sat. | | 861.28 | 861.27 | 861.26 |

^a^sNCM samples were subjected to identical processing conditions but without PI coating.

**Table S2. Summary comparison of cycle stability of our work with published polymer surface-modified sNCM cathodes in sulfide-based ASSLBs with the voltage 2.8-4.3 V vs. Li/Li^+^.**

| ***Ref.*** | **Coating** | **Cathode materials** | **Solid-state electrolytes** | **Cycle stability** | | |
| --- | --- | --- | --- | --- | --- | --- |
|  |  |  |  | **Rate** | **capacity retention** | **cycles** |
| **Our work** | PI | sNCM | Li_6_PS_5_Cl | 1 C | 83.6% | 400 |
| **6** | PEDOT:PSS | NCM262 | Li_6_PS_5_Cl | 1 C | 51.6% | 100 |
| **7** | No coating | sNCM811 | Li_10_SnP_2_S_12_ | 1 C | 64.5% | 100 |
| **8** | Ethyl cellulose | NCM811 | Li_6_PS_5_Cl | 1 C | 64% | 100 |
| **9** | cPAM | NCM811 | SCPE | 1 C | 75% | 100 |
| **10** | PAN | NCM811 | Li_6_PS_5_Cl | 1 C | 86.3% | 200 |
| **11** | EMG & butyl butyrate | NCM78 | Li_6_PS_5_Cl | 0.2 C | 85.1% | 300 |
| **12** | poly(tetrafluoroethylene-co-perfluoro(3-oxa-4-pentenesulfonic acid)) lithium salt | NCM712 | Li_6_PS_5_Cl | 0.5 C | 90% | 300 |
| **13** | PAA | NCM83 | Li_6_PS_5_Cl | 0.5 C | 92% | 200 |
| **14** | Decanoic acid | NCM85 | Li_6_PS_5_Cl | 0.5 C | 90.4% | 200 |
| **15** | sPPSLi/PVP | NCM95 | Li_6_PS_5_Cl | 0.1 C | 78.3% | 100 |
| **16** | PVBTA-TFSI | NCM83 | Li_6_PS_5_Cl | 0.1 C | 86% | 100 |
| **17** | PEG- PANI | NCM622 | Li_6_PS_5_Cl | 0.1 C | 88% | 100 |
| **18** | PDA@VGCF | NCM811 | Li_6_PS_5_Cl | 0.1C | 96% | 100 |

| **Table S3. XPS binding energies of key functional groups identified from peak fitting analysis for the cycled sNCM and sNCM@PI0.05 cathodes.**   \| **Species** \| **Assignment** \| **sNCM(eV)** \| **sNCM@PI0.05(eV)** \| \| --- \| --- \| --- \| --- \| \| **Li 1s** \| LiCl \| 56.30 \| 56.25 \| \| Argyrodite Li \| 55.49 \| 55.53 \| \| **O 1s** \| C-O \| 533.34 \| 533.33 \| \| C=O \| 531.80 \| 531.65 \| \| Lattice O \| 530.02 \| 529.97 \| \| **S 2p** \| P-S-Li \| 161.91 \| 161.95 \| \| P-S-P \| 163.68 \| 163.56 \| \| -S-S- \| 164.27 \| 163.96 \| \| SOx \| 168.81 \| -- \| \| **P 2p** \| PS_4_^3-^ \| 132.79 \| 132.93 \| \| P_2_S_x_ \| 133.78 \| 133.9 \|   **Table S4. Simulated results for Nyquist plot.** | | | | | | | |
| --- | --- | --- | --- | --- | --- | --- | --- | --- | --- | --- | --- | --- | --- | --- | --- | --- | --- | --- | --- | --- | --- | --- | --- | --- | --- | --- | --- | --- | --- | --- | --- | --- | --- | --- | --- | --- | --- | --- | --- | --- | --- | --- | --- | --- | --- | --- | --- | --- |
| **Number of cycles** | **sNCM** | | | | **sNCM@PI0.05** | | |
|  | ***R_b_*(Ω)** | ***R_f_*(Ω)** | ***R_CT_*(Ω)** | ***R_b_*(Ω)** | | ***R_f_*(Ω)** | ***R_CT_*(Ω)** |
| **1** | 31.5 | 21.2 | 111.4 | 41.5 | | 68.41 | 345.1 |
| **20** | 29.2 | 231.1 | 52.4 | 39.8 | | 53.5 | 345.9 |
| **40** | 29.2 | 264.4 | 55.7 | 34.6 | | 49.3 | 330.2 |
| **60** | 28.9 | 333.8 | 24.87 | 33.4 | | 53.2 | 258.2 |
| **80** | 28.5 | 321.9 | 82.8 | 33.7 | | 41.7 | 233.7 |
| **100** | 28.6 | 332.5 | 89.6 | 32.6 | | 40.2 | 214.6 |

**Table S5. Quantitative resistance values of the bare sNCM and sNCM@PI0.05 extracted from the DRT peak analysis during cycling.**

| **Number of cycles** | **sNCM^a^** | | | | | **sNCM@PI0.05^a^** | | |
| --- | --- | --- | --- | --- | --- | --- | --- | --- |
|  | ***R_b_*(Ω)** | ***R_f1_*(Ω)** | ***R_f2_*(Ω)** | ***R_CT_*(Ω)** | ***R_b_*(Ω)** | | ***R_f_*(Ω)** | ***R_CT_*(Ω)** |
| **1** | 5.5 | 12.3 | 30.9 | 13.6 | 8.0 | | 35.4 | 123.9 |
| **20** | 5.5 | 11.8 | 75.7 | 18.3 | 7.5 | | 29.4 | 125.4 |
| **40** | 5.7 | 12.1 | 91.3 | 17.4 | 6.8 | | 24.4 | 97.2 |
| **60** | 5.6 | 7.1 | 111.1 | 23.5 | 6.4 | | 27.8 | 110.7 |
| **80** | 5.6 | 8.5 | 107.3 | 40.4 | 6.3 | | 26.9 | 91.6 |
| **100** | 5.5 | 8.1 | 104.3 | 44.7 | 5.7 | | 26.8 | 107.3 |

^a^The exact resistance values (Ω) were calculated by integrating the area under the deconvoluted Gaussian peaks in the respective DRT profiles.

**References**

[1] Noh, H.-J.; Youn, S.; Yoon, C. S.; Sun, Y.-K. *J. Power Sources* **2013**, *233*, 121–130.

[2] a) Jia, P.; Liu, J.; Kong, J.; Hu, M.; Qi, N.; Chen, Z.; Xu, S.; Li, N. *Sep. Purif. Technol.* **2022**, *282*, 120044. b) Gao, G.; Xue, S.; Wang, L.; Wang, Y. *J. Membr. Sci.* **2023**, *686*, 122031.

[3] a) Sicklinger, J.; Metzger, M.; Beyer, H.; Pritzl, D.; Gasteiger, H. A. *J. Electrochem. Soc.* **2019**, *166*, A2322. b) Jung, R.; Morasch, R.; Karayaylali, P.; Phillips, K.; Maglia, F.; Stinner, C.; Shao-Horn, Y.; Gasteiger, H. A. *J. Electrochem. Soc.* **2018**, *165*, A132.

[4] Qi, K.; Wang, Y.; Dong, N.; Liu, B.; Tian, G.; Qi, S.; Wu, D. *Appl. Energy* **2022**, *320*, 119282.

[5] a) Cho, J.-H.; Park, J.-H.; Lee, M.-H.; Song, H.-K.; Lee, S.-Y. *Energy Environ. Sci.* **2012**, *5*, 7124–7131. b) Zhang, M.; Wang, L.; Wu, Y.; Liu, J.; Hu, Q.; Wang, X.; Xu, H.; He, X. *J. Energy Storage* **2023**, *72*, 108290. c) Wang, C.; Kang, Y.; Liu, B.; Lin, D.; Tian, G.; Qi, S.; Wu, D. *ACS Sustainable Chem. Eng.* **2025**, *13*, 7687–7697.

[6] Wu, F.; Liu, J.; Li, L.; Zhang, X.; Luo, R.; Ye, Y.; Chen, R. *ACS Appl. Mater. Interfaces* **2016**, *8*, 23095–23104.

[7] Liu, X.; Zheng, B.; Zhao, J.; Zhao, W.; Liang, Z.; Su, Y.; Xie, C.; Zhou, K.; Xiang, Y.; Zhu, J.; et al. *Adv. Energy Mater.* **2021**, *11*, 2003583.

[8] Zhang, J.; Zhong, H.; Zheng, C.; Xia, Y.; Liang, C.; Huang, H.; Gan, Y.; Tao, X.; Zhang, W. *J. Power Sources* **2018**, *391*, 73–79.

[9] Zheng, J.; Jiang, H.; Xu, X.; Zhao, J.; Ma, X.; Sun, W.; Liu, S.; Xie, W.; Chen, Y.; Xiong, S.; et al. *Nano-Micro Lett.* **2025**, *17*, 195.

[10] Huang, Y.; Tao, M.; Mo, L.; Zheng, L.; Su, D.; Jiang, J.; Pan, Q.; Hu, S.; Wang, H.; Li, Q.; et al. *Chem. Eng. J.* **2024**, *493*, 152525.

[11] Hong, S. B.; Jang, Y. R.; Kim, H.; Jung, Y. C.; Shin, G.; Hah, H. J.; Cho, W.; Sun, Y. K.; Kim, D. W. *Adv. Energy Mater.* **2024**, *14*, 2400802.

[12] Hong, S.-B.; Lee, Y.-J.; Kim, U.-H.; Bak, C.; Lee, Y. M.; Cho, W.; Hah, H. J.; Sun, Y.-K.; Kim, D.-W. *ACS Energy Lett.* **2022**, *7*, 1092–1100.

[13] Yi, M.; Li, J.; Wang, M.; Fan, X.; Hong, B.; Zhang, Z.; Wang, A.; Lai, Y. *J. Energy Chem.* **2024**, *89*, 137–143.

[14] Huang, Y.; Zhou, L.; Li, C.; Yu, Z.; Nazar, L. F. *ACS Energy Lett.* **2023**, *8*, 4949–4956.

[15] Shi, B.-X.; Weber, F.; Yusim, Y.; Demuth, T.; Vettori, K.; Münchinger, A.; Titvinidze, G.; Volz, K.; Henss, A.; Berger, R.; et al. *J. Mater. Chem. A* **2025**, *13*, 2600–2614.

[16] Shi, B.-X.; Yusim, Y.; Sen, S.; Demuth, T.; Ruess, R.; Volz, K.; Henss, A.; Richter, F. H. *Adv. Energy Mater.* **2023**, *13*, 2300310.

[17] Diao, H.; Jia, M.; Zhao, N.; Guo, X. *ACS Appl. Mater. Interfaces* **2022**, *14*, 24929–24937.

[18] Vuong, T. H. L.; Mastoi, N. R.; Nam, J. S.; Ran, W. T. A.; Ha, C.; Park, M.-S.; Kim, Y.-J. *Chem. Eng. J.* **2024**, *497*, 154534.
